# The Structural, Biological, and In-Silico Profiling of Novel Capryloyl Tetra-Glucoside and Aliphatic Ester Constituents from the *Abutilon indicum* Offers New Perspectives on the Treatment of Pain and Inflammation

**DOI:** 10.3390/plants11192583

**Published:** 2022-09-30

**Authors:** Shadma Wahab, Abdulrhman Alsayari, Abdullatif Bin Muhsinah, Dalia Almaghaslah, Anzarul Haque, Mohammad Khalid, Sulaiman Mohammed Alnasser, Faizul Azam, Md. Sarfaraj Hussain

**Affiliations:** 1 Department of Pharmacognosy, College of Pharmacy, King Khalid University, Abha 61421, Saudi Arabia; 2 Complementary and Alternative Medicine Unit, College of Pharmacy, King Khalid University, Abha 61421, Saudi Arabia; 3 Department of Clinical Pharmacy, College of Pharmacy, King Khalid University, Abha 61421, Saudi Arabia; 4 Department of Pharmaceutics, Buraydah College of Pharmacy and Dentistry, Buraydah P.O. Box 31717, Al-Qassim, Saudi Arabia; 5 College of Pharmacy, Department of Pharmacognosy, Prince Sattam Bin Abdul Aziz University, Al Kharj 11942, Saudi Arabia; 6 Department of Pharmacology and Toxicology, Unaizah College of Pharmacy, Qassim University, Unaizah 51911, Saudi Arabia; 7 Department of Pharmaceutical Chemistry and Pharmacognosy, Unaizah College of Pharmacy, Qassim University, Unaizah 51911, Saudi Arabia; 8 Lord Buddha Koshi Pharmacy College, Baijanathpur, NH 107, Saharsa 852201, Bihar, India

**Keywords:** *Abutilon indicum*, isolation, anti-inflammatory, analgesic, molecular docking

## Abstract

*Abutilon indicum L.* (Malvaceae), more often referred to as Peeli booti, Kanghi, and Kakhi, is a perennial shrub found in many countries of Asia. Traditionally, this plant is used as a diuretic to treat inflammation, discomfort, urethral infections, and gout. Inflammation and pain are key topics of interest for researchers throughout the globe, since they are linked to almost every illness that could affect humans or animals. The present study was conducted to isolate the phytoconstituents from the methanolic extract of *Abutilon indicum* collected from the Bihar state Koshi river belt in India, and to evaluate the isolated phytoconstituents’ ability to reduce nociception and inflammation. Furthermore, molecular docking was performed to investigate the molecular interaction profile, with possible therapeutic targets for anti-inflammatory medicines. *A. indicum* methanolic extract yielded two novel phytocompounds identified as 5′-hydroxyhexyl n-hexadecanoate (AB-01) and n-octanoyl-β-D-glucopyranosyl-(2′-1′′)-β-D-glucopyranosyl-(2′′-1′′′)-β-D-glucopyranosyl-(2′′′-1′′′′)-β-D-glucopyranoside (AB-05), together with three previously recognized phytocompounds such as ester glucoside. All isolated molecules were tested for the efficacy of analgesic and anti-inflammatory characteristics at doses of 5 and 10 mg/kg body weight. The isolated compound’s molecular interaction profile with anti-inflammatory drug targets cyclooxygenase-2 and tumor necrosis factor-alpha possessed high potential energy in molecular docking. These findings may aid in developing anti-inflammatory and analgesic drugs from *A. indicum.*

## 1. Introduction

*Abutilon indicum* L. (Malvaceae), known locally as Peeli booti, Kanghi and Kakhi, is a perennial shrub found in many countries of Asia [1]. Ethnobotanical surveys have found that nearly all plant parts are healthy and have been used for centuries to treat various diseases. Roots and leaves are used as diuretics and demulcents to treat urethritis and chest infections, and for local analgesia and inflammation reduction in cases of toothache, sore gums and of the bladder. *A. indicum* decoction is traditionally used in different folklore preparations for treating earache, toothache, and other inflammatory pain disorders. The fruits and seeds have been used to treat various ailments, including febrile illnesses such as piles, chronic cystitis, and gonorrhea [2,3]. Furthermore, for jaundice, gout, asthma, tuberculosis, coughs, ulcers, diarrhea and managing various endocrine disorders, including diabetes and infertility, it has been used traditionally [4,5]. Phytochemical analysis of *A. indicum* shows the presence of active polyphenols, steroids, tannins, terpenoids, saponins, glycosides, flavonoids (gallic acid, β-sitosterol, geraniol, caryophyllene) [6], two sesquiterpene lactones (alantolactone, isoalantolactone) [7], and essential oils (β-amyrin, eudesmol, eugenol, geraniol, and caryophyllene), gossyptin-7-glucoside, cyanidin-3-rutinoside, and gossypetin-8-glucoside [8]. Our previous work suggested that the methanolic extract of *A. indicum* contains various phytoconstituents such as gallic acid, quercetin, Stigmasterol and lupeol [9,10]. Pharmacological evaluation of *A. indicum* validated hepatoprotective [7], anti-oxidant [8], anti-inflammatory [11], cytotoxic and anti-microbial [12,13], hypoglycemic [14], anti-diarrheal [15], anti-ulcer [16], anti-proliferative [16], anti-diabetic [4], analgesic [17], anti-arthritic [18], anti-asthmatic [19], and immunomodulatory activities [20]. In a separate study, the methanolic extract was reported to have anti-arthritic activity [18].

Inflammation and pain have emerged as significant areas of interest for investigators worldwide, due to their connections to almost every disease that may affect humans or animals. Furthermore, the potentially harmful effects of non-steroidal anti-inflammatory medicines (NSAIDs) and opioids have forced researchers to look for alternative treatments with less adverse effects [21,22,23]. Therefore, the effectiveness of traditionally useful medicinal herbs as anti-inflammatory and analgesic agents must be thoroughly examined scientifically, utilizing the latest experimental tools and methodologies. In developing drugs from natural products, computational molecular modelling has become an essential topic in recent years. Drug development from natural products is facilitated by in silico drug design techniques. Computational modelling reveals molecular recognition mechanisms underpinning disease-related target macromolecule interactions with drug-like compounds [24,25].

However, most of the pharmacological work reported on the extract of *A. indicum. Despite* its extensive use in traditional medicine in the treatment of various pain and inflammatory disorders in the eastern area of India, little work has been carried out on the isolated constituents of *A. indicum* from a scientific standpoint. In light of this, the current investigation was carried out to isolate the phytoconstituents from the methanolic extract of *Abutilon indicum* and to determine the isolated phytoconstituent capacity to inhibit nociception and inflammation. In addition, molecular docking was used to study the molecular interaction profile with various potential therapeutic targets for anti-inflammatory medications.

## 2. Materials and Methods

### 2.1. Collection of Plant Material

For research and experiment, in January 2016, whole *A. indicum* plants were harvested from the field region of Baryahi, Saharsa District, Bihar, India. The plant specimen was certified as authentic by Professor (Dr.) Anjani Kumar Sinha of M. L. T. Saharsa College in Saharsa, Bihar. A voucher specimen number (#SHC 56/01/2016) for future reference has been deposited in the herbarium of the Botany Department of M. L. T. Saharsa College, Saharsa- 852201.

### 2.2. Extraction and Isolation

An amount of 4.8 kg of dried *A. indicum* plant material was pulverized and completely covered with methanol (99.8% *v*/*v*) in a Soxhlet apparatus for 72 h. The methanolic extract was concentrated at low pressure to produce a dark brown viscous mass (181.0 g). Only a small portion of the *A. indicum* extract was chemically tested to determine the presence of various phytochemicals. The concentrated extract (80 g) of the drug was placed in a China dish and continuously cooked in a water bath by progressively adding methanol (100 mL) in small parts with constant stirring until the desired consistency was achieved. A weighed quantity of silica gel for column chromatography (Merck, Mumbai, India) was gently added with continuous mixing with a steel spatula until the entire methanolic solution of plant extract was adsorbed onto silica gel particles. It was air-dried and rubbed between hands to break up the larger lumps before passing through a sieve (No. 8), to achieve uniform particle size.

A 16-mm-diameter 5-foot-tall column was obtained, cleansed, and dried. Non-absorbent cotton wool was used to plug the lower end of the column. The column was fastened and placed on a stand in a vertical position. Petroleum ether (b.p. 60–80 °C) was then half-filled into the column. The dried plant extract slurry was put into the column and then eluted sequentially with different solvents of increase polarity. Silica gel (for the column, 60–120 mesh, Merck, Mumbai, India) was then poured in small parts and allowed to settle down. The column was developed and eluted with a series of different solvents in various combinations, including petroleum ether, petroleum ether: chloroform (9:10, 3:1, 1:1, 1:3 *v*/*v*), chloroform, chloroform: methanol (99:1, 98:2, 97:3, 24:1, 19:1, 9:1, 3:1, and 1:1, 1:3 *v*/*v*), chloroform. Different ratios were taken and tested using TLC to assess the homogeneity (Silica gel G precoated TLC plates, Merck, Mumbai, India). Fractions with identical Rf values were combined and crystallized with solvents. Their spots were visualized by exposing them to iodine vapors, UV radiations (254 and 366 nm) and spraying with 0.1% anisaldehyde sulfuric acid as spraying reagents. The separated phytochemicals were then recrystallized to obtain pure compounds. The compound obtained from the column was crystalline pure. It showed clear Rf, melting point, and UV lambda max values. The purity of the compound was also checked by the hydrolysis of compounds and the Rf value of individual components.

### 2.3. General Experimental Procedures

Methanol (Merck, Mumbai, India) was used as a solvent in the Shimadzu-120 double beam spectrophotometer to determine Ultraviolet spectra. Shimadzu FTIR-8400 was used to detect and document IR spectra in KBr pellets. Scanning of ^1^H NMR (300 MHz) and ^13^C NMR (75 MHz) spectra was carried out using the Bruker spectrospin NMR apparatus exploiting TMS as the internal standard. EIMS at 70 eV with a Jeol D-300 device (Jeol, USA) was used for scanning, and silica gel (60–120 mesh, Merck, Mumbai, India) was used for column chromatography. For thin-layer chromatography, silica gel G-coated TLC plates manufactured by Merck in Mumbai, India, were used. For thin-layer chromatography, silica gel G-coated TLC plates (Merck, Mumbai, India) were employed. Based on the dried plant material acquired for extraction (4.8 kg), an estimate of the % yields of the isolated compounds were determined.

### 2.4. Animals

Adult Swiss albino mice of both sexes, weighing between 25 and 30 g, were used for the analgesic activity study, while healthy Wistar albino rats of both sexes, weighing around 140 and 160 g, were used for the anti-inflammatory activity study. They were kept in clean, sterilized polypropylene cages at ambient temperature (21 ± 2 °C) and under a dark/light control for 12 h. Additionally, they were fed commercial pellets and had access to water *ad libitum*. Mice were randomly assigned to groups and kept in isolation for a week to allow for sociability and acclimation to their new surroundings and caretakers before the experiments. The research was approved (Phar-02/2018) and conducted in accordance with the recommendations of the Institutional Ethical Committee of the Faculty of Pharmacy at Misurata University in Misurata, Libya.

#### 2.4.1. Safety Profile Study

An acute toxicity study was conceded for the assurance of LD_50_ by carrying out the plan (Annexure 2d) of CPCSEA, OECD guideline No. 423. Swiss albino mice of each sex were divided into seven groups, with six animals in each group. Isolated compounds AB-01, AB-02, AB-03, AB-04 and AB-05 from a methanolic extract of *A. indicum* at various dose levels of 50, 100, and 150 mg/kg were administered orally as a single dose to mice. The animals were observed periodically for indications of toxicity and death for 24 h, and afterwards consistently for 14 days [26].

#### 2.4.2. Administration of Drugs

In all the experimental animals, the isolated chemicals AB-01, AB-02, AB-03, AB-04, and AB-05 were given at 05 and 10 mg/kg dosages. In the hot plate approach, Indomethacin 10 mg/kg was used as a typical anti-inflammatory medicine, and Tramadol 0.1 mL (40 mg/kg. s.c.) was employed as a pain-inhibiting agent. In mice with acetic acid-induced writhing, 5 mg/kg of diclofenac was used as a painkiller. To obtain the necessary dosage on a bodyweight basis (mg/kg) of the animal, all the test and standard drugs were mixed into an emulsion using 0.3 percent carboxy methyl cellulose (CMC). This emulsion was then delivered orally using a safety needle with a ball-finished tip [27].

#### 2.4.3. Carrageenan-Induced Rat Hind Paw Edema

Acute inflammation was incited by the injection of 0.1 mL of 1% freshly prepared suspension of carrageenan in normal saline into the sub-plantar region of the right hind paw of all groups of animals. At 1, 2, 3, 4, and 5 h intervals, the volume of the injected paws was measured using a plethysmometer. The animals were premedicated with the vehicle (0.3% CMC p.o.), isolated compounds AB-01, AB-02, AB-03, AB-04 and AB-05 at doses 05 and 10 mg/kg, and the standard drug indomethacin (10 mg/kg) 1 h before the carrageenan challenge [28]. The percentage inhibition of inflammation was intended to be consistent with the following formula: % inhibition = 100 (1 − Vt/Vc), where ‘Vc’ stands for the inflammation volume in the control and ‘Vt’ for the inflammation volume in the group treated with tested drugs.

#### 2.4.4. Analgesic Activity

The isolated compounds AB-01, AB-02, AB-03, AB-04, and AB-05 were tested for their analgesic effectiveness using a chemical approach (an acetic acid-induced writhing response) and a thermal approach method (hot plate reaction time).

##### Hot Plate Test

The pain-relieving (analgesic) test was carried out with an Eddy’s hot plate, maintained at a temperature of 55 ± 1 °C. The basal response time of all mice towards thermal heat was verified first. They were then treated with the vehicle (0.3% CMC p.o.) along with the isolated compounds AB-01, AB-02, AB-03, AB-04 and AB-05 at doses of 05 and 10 mg/kg and the standard medication Tramadol 0.025 mL (10 mg/kg. s.c). Following an hour of testing and standard medication dosing, the mice in all groups were placed individually on the hot plate, which was maintained at 55 °C. The snapshot of time required in seconds for paw licking or bouncing was noted as reaction time. A remove phase of 30 s was maintained to avoid harm to the paw. The pain inhibition percentage (PIP) was determined by the accompanying equation [29]:

Pain inhibition percentage (PIP) = ((T_1_ − T_0_)/T_0_) × 100

where T_1_ is post-drug latency, and T_0_ is pre-drug latency time.

##### Acetic Acid-Induced Writhing Test

The animals were pre-medicated with the vehicle (0.3% CMC p.o.), AB-01, AB-02, AB-03, AB-04 and AB-05 at doses 05 and 10 mg/kg, and the standard medication diclofenac (5 mg/kg). Acetic acid (1% *v/v*) in doses of 1 mL/kg body weight was injected intra-peritoneally in all the groups of animals 1 h after dosing of the test and standard drugs. Writhing was recorded by counting the number of writhes following the injection of acetic acid for a time of 30 min. A writhe was identified as hind limb abdominal constriction and full extension [29].

### 2.5. ADMET Analysis

Chemical Scheme of all the test compounds (AB 01 to AB 05) were drawn up using Chemdraw Ultra 12.0 and converted to mol files. These files were then imported to Maestro 9.0 workspace and subjected to ligand preparation. Once the ligands had been prepared, these were subjected to the Qikprop tool for absorption, distribution, metabolism, excretion and toxicology (ADMET) profiling [30,31].

### 2.6. Molecular Docking

#### 2.6.1. Ligand Preparation

Molecular docking methods are frequently employed to compute the binding affinities of a range of ligands [30,32,33,34]. The structure of the isolated molecules was drawn in a chem sketch and shown to possess high energy. Therefore, an energy minimization approach was applied to optimize the molecule structures. Energy minimization of the ligands was carried out using the ‘Minimize Ligands’ module from Biovia Discovery Studio, employing the CHARMm forcefield and Smart Minimizer algorithm for 2000 steps. The smart Minimizer method made the original structure different from the minimal structure. All the minimized ligands were converted to pdbqt before the docking procedure [33,35].

#### 2.6.2. Protein Preparation

The TNF-alpha receptor protein (PDB ID: 2AZ5_A) was retrieved from the RSCB protein data bank (http://www.rcsb.org/) (accessed on 11 April 2022). Each protein was cleaned of water molecules and co-crystal ligands. To produce pdbqt files, the protein was assigned polar hydrogens, Kollman charges, solvation parameters, and fragmental volumes using the Graphical User Interface tool AutoDock Tools (ADT) Version 4.2. To generate a grid around the protein, ADT was used to create a grid around the binding pocket. A grid map was created using AutoGrid and a grid box. The grid dimensions were set at −19.163, 74.452, and 33.837 in x, y, and z coordinates in the binding pocket with a grid spacing of 0.375. The grid size was set to 78, 60, and 72 in x, y, and z points in the binding pocket. Using the grid box properties specified in the configuration file, AutoDock Vina was used to dock proteins and ligands. Throughout the docking process, proteins were labelled rigid, whereas ligands were labeled flexible. The findings with a positional root-mean-square deviation (RMSD) less than 1.0 were clustered together and represented by the binding free energy result with the lowest value. The ligand conformation with the lowest binding energy or affinity was selected and fitted with the receptor structure for intra-molecular interaction studies [11,36]. The ‘View Interaction’ protocol from the Discovery Studio (DS) [Dassault Systems, BIOVIA Corp., San Diego, CA, USA, v 20.1] application was used to conduct the interaction analysis in this study.

### 2.7. Statistical Analysis

Graph Pad Prism V2.01 was used to carry out a one-way variance analysis, followed by Dunnett’s post-hoc test (GraphPad Software, Inc., San Diego, CA, USA). Experiments were performed in triplicate and repeated three times. The data were expressed as the mean ± standard error of the mean, and *p* < 0.05 and <0.01 were considered statistically significant.

## 3. Results

### 3.1. Structural Profiling of Isolated Phytoconstituents

#### 3.1.1. Compound (AB-01)- 5′ Hydoxyl Palmitate

A colorless crystalline mass of AB-01 was obtained when the column was eluted with a mixture of chloroform and methanol in the proportion of 19:1. This was recrystallized with chloroform: methanol in a ratio of 1:1 that yielded 95 mg mass of yield 0.12%. The R_f_ value 0.39 was received for the CHCL_3_ -MeOH in a ratio of 19:1; UVλ_max_ (MeOH)-207 nm, m.p. 105–107 °C. KBr-IR ν_max_ was shown 1049, 1224,1401, 1654, 1722, 2845, 2927, 3410 cm^−^^1^. ^1^H NMR of MeOD-0.88 (6H, m, Me-16, Me-6′), 1.28 (28H, brs,14 × CH2), 1.55 (2H, m, CH_2_), 1.32 (4H, M, CH_2_), 2.56 (2H, t, J = 7.5 Hz, H_2_-2), 3.50 (2H, t, J = 7.2 Hz, H_2_-1′), 3.64 (1H brm, w _1/2 =_ 11.2 Hz, H-5′. ^13^CNMR (CDCl_3_), 14.21 (Me-14), 18.16 (Me-1′), 22.48 (CH_2_), 30.23 (13xCH_2_), 30.42 (CH_2_), 33.57 (CH_2_), 38.32 (CH_2_), 64.39 (C-1′), 73.90 (C-5′), 173.41 (C-1). +ve ESI-MS *m/z* (rel. int.) -356 [M]^+^(C_22_H_44_O_3_) (2.1), 311 (2.3), 239 (3.8), 117 (4.6).

#### 3.1.2. Compound (AB-02)- Capryloyl Diglucoside

The column was then eluted using a mixture of methanol and chloroform (1:19) that yielded a colorless mass of AB-02. This was recrystallised with methanol, and a 103 mg mass of 0.11% yield was obtained. UV λ_max_ (MeOH): 210 nm; R_f_ 0.42 (MeOH: CHCl_3_-1:19); m.p. 115–118 °C; (KBr) IR ν_max_: 779, 867, 1059, 1231, 1403, 1721, 2852, 2936, 3265, 3389, 3450 cm^−1^. ^1^H NMR (MeOD)- 0.88 (3H, t, J = 6.3 Hz, Me-8); 1.29 (8H, brs,4 × CH_2_), 1.39 (2H, m, CH_2_), 2.56 (2H, t, J = 7.2 Hz, H_2_-2), 3.10 (2H, brs, H_2_-6′′), 3.34 (2H, brs, H_2_-6′), 3.48(1H, m, H-4′′), 3.57 (1H, m, H-4′), 3.64 (1H, m, H-3′′), 3.67 (1H, m, H-3′), 3.70 (1H, m, H-2′′), 3.74 (1H, m, H-5′′), 3.78 (1H, m, H-5′), 3.83 (1H, m, H-2′), 3.99 (1H, d, J = 7.1 Hz, H-1′′), 4.04 (1H, d, J = 7.2 Hz, H-1′). ^13^C NMR (MeOD): 14.21(ME-8); 22.63 (CH_2_), 30.12 (4 × CH_2_), 34.82 (CH_2_), 62.74 (C-6′′), 64.55 (C-6′), 66.04 (C-4′′), 69.53 (C-4′), 71.39 (C-3′′),71.97 (C-3′), 71.97 (C-3′), 73.99 (C-2′′), 76.92(C-5′′), 77.64 (C-5′), 83.41(C-2′), 99.33(C-1′′), 103.29 (C-1′), 172.06 (C-1). +ESI MS *m/z* (rel.int): 468[M]^+^ (C_20_H_36_O_12_), 326 (12.5), (5.3), 163 (47.8), 127 (100), 143 (32.1).

**Hydrolysis of AB-02:** Compound 2 (15 mg) was dissolved in ethanol (5 mL). Concentrated hydrochloric acid (2 mL) was then added, and the reaction mixture was heated for 1 h in a steam bath. Following this, it was chilled and extracted using petroleum ether, equivalent to a reference capric acid sample, on a TLC plate developed with petroleum ether to separate the capric acid. After separating the fatty acids using n-butanol-acetic acid water and a reference sugar sample, the residue was concentrated and chromatographed on a silica gel TLC plate (4:1:5, top layer). The sugar was revealed to be D-glucose, according to *R_F_*. 0.12.

#### 3.1.3. Compound (AB-03)- Capryl Diglucoside

Compound AB-0**3** was eluted from the column with a mixture of chloroform and methanol (9:1), resulting in a colorless mass. It was then recrystallised with methanol, which eventually yielded 118 mg (0.16% yield). The R_f_ 0.51 (CHCl_3_-MeOH, 9:1) and m.p. 120–122 °C. The UV λ_max_ (MeOH) was noted at 270 nm. Using KBr- IR ν_max_ was recorded: 779, 1059, 1240, 1402, 1638, 1721, 2930, 3261, 3389, 3425 cm^−1^. ^1^H NMR (MeOD)-δ 0.86 (3H, t, J = 6.3 Hz, Me-10); 1.52 (2H, m, CH_2_, 1.29 (12H, brs, 6 × CH_2_), 2.23 (2H, t, J = 7.2 Hz, H_2_-2), 3.30 (1H, d, J = 3.0 Hz, H_2_-6′′ b), 3.32 (1H, d, J = 3.0 Hz, H_2_-6′′ a), 3.35 (2H, brs, H_2_-6′), 3.46 (1H, m, H-4′′), 3.50 (1H, m, H-4′), 3.58 (1H, m, H-3′′), 3.62 (1H, m, H-3′), 3.68 (1H, m, H-2′′), 3.71 (1H, m, H-5′′), 3.78 (1H, m, H-5′), 6.5 Hz, H-2, 3.83 (1H, dd, J = 7.3, ′), 4.01 (1H, d, J = 7.1 Hz, H-1′′),4.06 (1H, d, J = 7.3 Hz, H-1′). ^13^C NMR (MeOD)-δ 22.61 (CH_2_), 14.23 (Me-8); 29.48 (5 × CH_2_), 30.94 (CH_2_), 34.91 (CH_2_), 64.33 (C-6′′), 64.66 (C-6′), 66.01 (C-4′′), 69.51 (C-4′), 71.38 (C-3′′), 71.96 (C-3′), 73.97 (C-2′′), 76.89 (C-5′), 77.65 (C-5′), 83.39 (C-2′), 99.33 (C-1′′), 103.27 (C-1′), 170.16 (C-1). + Ve ESI MS *m/z* (rel.int)- 497 [M+H]^+^ (C_22_H_41_O_12_), (7.25), 155 (16.5), 163 (20.8), 325 (18.3), 341 (12.2).

**Hydrolysis of AB-03:** Hydrochloric acid (conc.) was added to the reaction mixture with Compound **3** (10 mg), and the reaction took place for one hour in a hot steam bath. Caprylic acid was isolated by using petroleum ether as a solvent. To chromatograph the fatty acid residue over the developing solvent system of n-butanol, acetic acid, and water (4:1:5), silica gel TLC plates with a standard sample of sugars were used. D-glucose, *R_F_* 0.12, was identified as the sugar.

#### 3.1.4. Compound (AB-04)- Palmityl Diglucoside

Crystals of AB-04, recrystallized from chloroform: methanol (l:1), were produced by further eluting the column with chloroform: methanol solvents (9:1). The yield was found to be 206 mg in terms of % yield 0.28. The R_f_ 0.72 (CHCl_3_-MeOH, 9:1) and m.p. were reported to be 105–110 °C. UV λ_max_ (MeOH) was shown at 217 nm. IR ν_max_ (KBr)- 779, 1059, 1228, 1402, 1654, 1725, 2845, 2930, 3256, 3397, 3448 cm^−1^. ^1^H NMR (MeOD)- 0.88 (3H, t, J = 6.2 Hz, Me-16); 1.29 (24H, brs, 12 × CH_2_), 1.60 (2H, m, CH_2_), 2.31 (2H, t, J = 7.2 Hz, H_2_-2), 3.30 (1H,d, J = 6.0 Hz, H_2_-6b), 3.32(1H, d, J = 6.0 Hz, H_2_-6a), 3.35 (2H, brs, H_2_-6), 3.49 (1H, m, H-3′), 3.58 (1H, m, H-3′), 3.61 (1H, m, H-4′′), 3.64 (1H, m, H-4′), 3.74 (1H, m, H-2′′), 3.78 (1H, m, H-5′′), 3.83 (1H, m, H-5′), 3.96 (1H, m, H-2′), 4.01 (1H, d, J = 7.0 Hz, H-1′′), 4.04 (1H, d, J = 7.2 Hz, H-1′). ^13^C NMR (MeOD)- 14.61 (Me-16); 22.55 (CH_2_), 30.03 (11 × CH_2_), 30.85 (CH_2_), 33.88 (CH_2_), 62.90 (C-6′′), 64.32 (C-6′), 64.66 (C-4′′), 66.02 (C-4′), 71.37 (C-3′), 71.95 (C-3′), 73.97 (C-2′′), 76.89 (C-5′′), 77.63(C-5′), 83.39 (C-2′), 99.32 (C-1′′), 103.27 (C-1′), 171.52 (C-1). +Ve ESI MS *m/z* (rel.int)-581 [M+H]^+^ (C_28_H_53_O_12_) (8.4), 163 (24.1), 239 (14.8), 255 (33.2).

**Hydrolysis of AB-04:** The reaction mixture containing Compound 4 (10 mg) was treated with ethanol (5 mL) and concentrated HCI (2 mL) for one hour in a hot steam bath. Palmitic acid was separated from the reaction mixture using chloroform extraction, and the co-TLC findings were then compared to these. After separating the fatty acid, it was concentrated and chromatographed on silica gel TLC. A reference sample of the sugar utilizing the top layer of n-BuOH-AcOH-H2O, 4:1:5 was used. The fatty acid was then separated. D-glucose was identified as the sugar (R_f_ 0.12) and was separated.

#### 3.1.5. Compound (AB-05)- Capryloyl Tetraglucoside

Column elution with methanol: chloroform in a ratio of (1:9) gave a crystalline mass of a light brown color AB-05, which was recrystallised again from methanol that yielded a 145 mg mass of 0.23% yield. The R_f_ 0.63 (CHCl_3_-MeOH, 9:1) and m.p. was found to be 125–126 °C. UV λ_max_ (MeOH) ranged at 206 nm. The KBr- IR ν_max_ values were 779, 1059, 1240, 1402, 1639, 1723, 2931, 3265, 3397, 3425 cm^−1^. ^1^H NMR (MeOD): δ 0.86 (3H, t, J = 6.3 Hz, Me-8); 1.25 (8H, brs, 4 × CH_2_), 1.52 (2H, m, CH_2_), 2.51 (2H, m, H_2_-2), 3.29 (2H, d, J = 6.1 Hz, H_2_-6′′′′), 3.31 (4H, d, J = 6.5 Hz, H2-6′′, H2-6′′′), 3.34 (2H, d, J = 6.2 Hz, H2-6′), 3.46 (1H, m, H-4′′′′), 3.48 (1H, m, H-4′′′), 3.52 (1H, m, H-4′′), 3.57 (1H, m, H-4′), 3.61 (1H, m, H-3′′′′), 3.64 (1H, m, H-3′′′), 3.69 (1H, m, H-3′′), 3.78 (1H, m, H-3′), 3.78 (1H, m, H-2′′′′), 3.82 (2H, m, H-5′′′, H-5′′′′), 3.84 (2H, m, H-5′, H-5′′), 3.87 (1H, m, H-2′′′), 3.93 (1H, H-2′′), 3.96 (1H, m, H-2′), 4.00 (1H, d, J = 7.3 Hz, 1 + -1′′′′), 4.02 (1H, d, J = 7.0 Hz, H-1′′′), 4.04 (1H, d, J = 7.2 Hz, H-1′′), 4.06 (1H, d, J = 7.1 Hz, H-1′). ^13^C NMR (MeOD): δ 14.27 (Me-8), 22.48 (CH_2_), 29.86 (4 × CH_2_), 33.13 (CH_2_), 62.62 (C-6′′′′), 62.63 (C-6′′′), 62.65 (C-6′′), 63.81 (C-6′), 64.62 (C-4′′′′), 65.13 (C-4′′′), 65.90 (C-4′′), 69.41 (C-4′), 71.28 (C-3′′′′), 71.87 (C-3′′′), 73.04 (C-3′′), 73.84 (C-3′), 74.89 (C-2′′′), 76.31 (C-5′′′′), 76.74 (C-5′′′), 77.51 (C-5′′), 77.99 (C-5′), 83.18 (C-2′′, C-2), 84.24 (C-2′), 98.16 (C-1′′′′), 99.33 (C-1′′′), 103.20 (C1′′), 105.89 ((C-1′), 171.13 (C-1). +ve ESI MS *m/z* (rel.int): 792[M]^+^ (C_32_H_56_O_22_) (6.2), 629 (7.0), 503 (14.8), 487 (6.2), 467(4.3), 305 (15.1), 289 (6.7), 179 (13.6), 163 (9.2), 143 (100), 127 (24.3).

**Hydrolysis of AB-05:** A steam bath was used to heat a combination of 15 mg of Compound 5 for 1 h, using conc. HCL 2 mL and ethanol 5 mL. Petroleum ether was employed to extract capric acid from the reaction mixture, and co-TLC results were equivalent. The reaction mixture was concentrated, and the standard sugar was chromatographed on silica gel TLC with n-butanol-AcOH-H_2_O in a ratio of 4:1:5 as the developing solvent system. D-glucose, R_F_ 0.12, was identified as the sugar.

### 3.2. Safety Profile Study

It was seen that the dosing of isolated compounds AB-01, AB-02, AB-03, AB-04 and AB-05 up to 150 mg/kg orally to the mice did not incite drug-related harmfulness (toxicity) and mortality. The results revealed the mice endured the drug well and showed normal behavior up to 150 mg/kg orally. All animals were vigilant, with normal spruce, touch, and pain response, and there was no indication of accommodation or vocalization.

### 3.3. Carrageenan-Induced Rat Paw Edema

**Figure 1:**
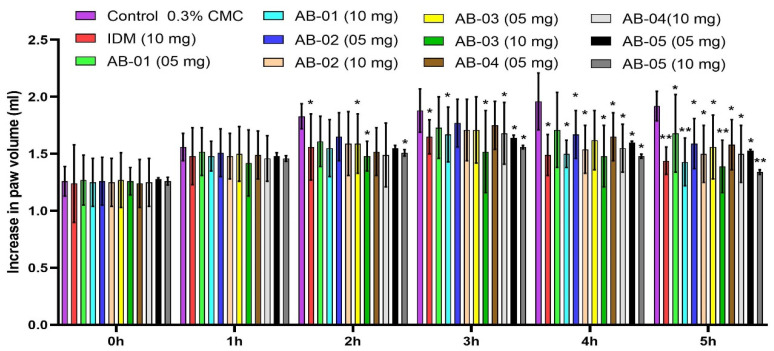
Effect of isolated compounds AB-01, AB-02, AB-03, AB-04 and AB-05 and Indomethacin (IDM) on carrageenan-induced rat paw edema. Each value is expressed in Mean ± S.E.M. one-way ANOVA followed by Dunnett’s test, *p*: * *p* < 0.05 and ** *p* < 0.01, compared with respective control group.

The anti-inflammatory effect of isolated compounds AB-01, AB-02, AB-03, AB-04 and AB-05 at doses of 5 & 10 mg/kg and Indomethacin at a dose of 10 mg/kg on carrageenan-disturbed rat paw is displayed in Figure 1. The anti-inflammatory effects of all tested drugs were recorded from 60 min after the carrageenan challenge. Paw edema in rats reached its highest at 4 h following the carrageenan challenge, and animals treated with Indomethacin showed a significant (*p* < 0.05) decrease in paw volume of rats from 2 h. Treatment with Indomethacin and AB-01, AB-02, AB-03, AB-04 and AB-05 at 10 mg/kg showed a significant reduction (*p* < 0.05) of paw volume in rats incited by carrageenan. The impact of AB-01 and AB-05 at a portion of 10 mg/kg was favored in lessening the swollen paw volume, rather than a higher portion of Indomethacin (10 mg/kg).

### 3.4. Hot Plate Test

**Figure 2:**
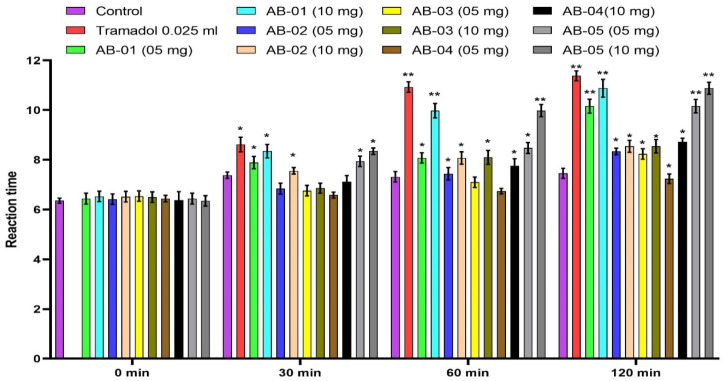
Effect of isolated compounds AB-01, AB-02, AB-03, AB-04, AB-05 and Tramadol (Standard) on the reaction time of mice exposed to the hot plate. Each value is expressed in Mean ± S.E.M. One-way ANOVA followed by Dunnett’s test, *p*: * *p* < 0.05 and ** *p* < 0.01, compared with respective control group.

AB-01 and AB-05 at a dose of 10 mg/kg and Tramadol significantly (*p* < 0.01) augmented the response time of the animals towards the thermal source at various periods (Figure 2). The hot plate test of isolated compounds AB-01, AB-02, AB-03, AB-04 and AB-05 (at dose 10 mg/kg p.o.) and Tramadol (10 mg/kg. s.c) displayed a pain inhibition percentage (PIP) of 71.45%, 36.78%, 66.73%, 44.93%, 74.79% and 79.21%.

### 3.5. Acetic Acid-Induced Writhing Methods

**Figure 3:**
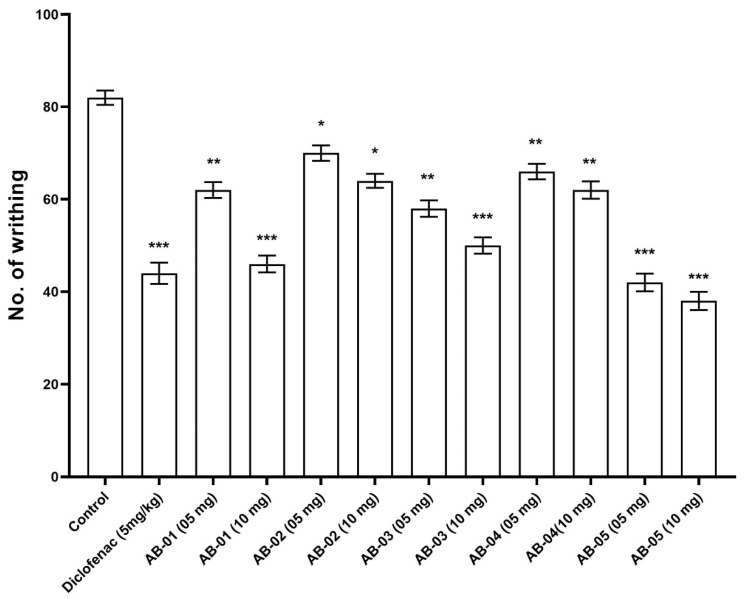
Effect of isolated compounds AB-01 to AB-05 and Diclofenac (Standard) on acetic acid-induced writhing in mice. Each value is presented as Mean ± S.E.M. One-way ANOVA followed by Dunnett’s test, *p*: * *p* < 0.05, ** *p* < 0.01 and *** *p* < 0.001, compared with respective control group.

The acetic acid-induced writhing assay was used to evaluate the analgesic efficacy of isolated compounds AB-01 to AB-05 at dosages of 5 and 10 mg/kg, and Diclofenac (5 mg/kg). The injection of acetic acid caused abdominal constriction and a contraction of the hind limbs, although AB-01, AB-05, and Diclofenac all substantially (*p* < 0.001) reduced this response (Figure 3). The abdominal constriction was lowered to 46.17%, 64.82%, 50.42%, 62.19% and 38.19% with compounds AB-01, AB-02, AB-03, AB-04 and AB-05, respectively, at 10 mg/kg. Diclofenac lowered abdominal constriction by 44.23% at a 5 mg/kg dose.

### 3.6. ADMET Analysis

ADMET analysis showed that compounds AB 01, AB 02 and AB 03 had a molecular weight below 500; they might be considered drug candidates. In contrast, compounds AB 04 and AB 05, having a molecular weight of more than 500, are poor candidates for drug development. The number of rotational bonds in all the compounds appeared to be more than the acceptable range. With the exception of compound AB 01, all compounds had more than the required hydrogen bond donors, while with the exception of compound AB 05, the remaining compounds had hydrogen bond acceptors within the specified range. With the exception of compound AB 02, all the compounds had partition coefficients within the range. The ADMET analysis result is included in Table 1.

### 3.7. Molecular Docking

**Figure 4:**
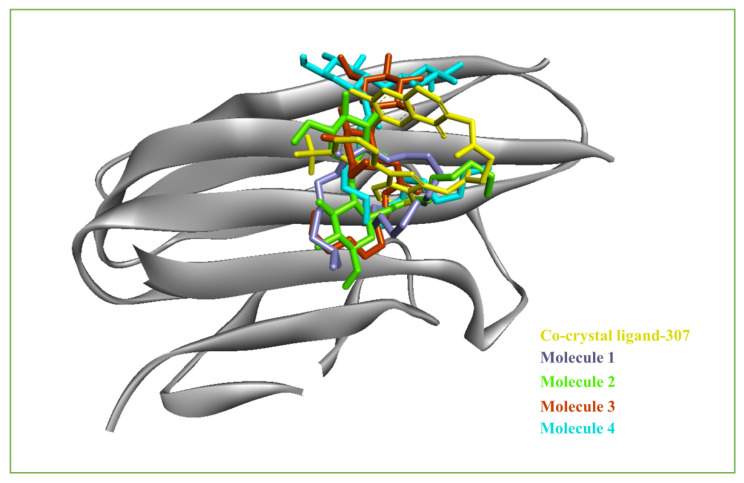
Superimposed conformations of the docked molecules and co-crystal ligand- 307 in the binding pocket of 2AZ5_A. The docked molecules are shown in the sticks, and the protein is shown in cartoon representation using Biovia Discovery Studio Visualizer.

**Figure 5:**
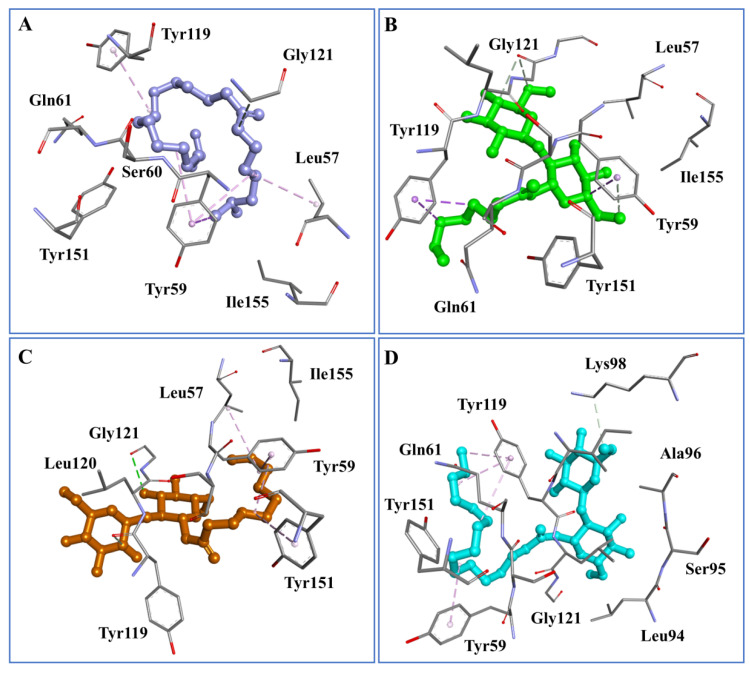
Docked complexes of Molecule 1 (**A**), Molecule 2 (**B**), Molecule 3 (**C**), and Molecule 4 (**D**) in the binding pocket of TNF-alpha (PDB ID: 2AZ5_A). The interacting residues of the protein are shown in the stick view, whereas molecules 1–4 are shown in the ball and stick. The images were drawn in Biovia Discovery Studio Visualizer.

The isolated constituents Ab-01 to AB-05 were docked with receptors known to be related to inflammatory pathways. These receptors included cyclooxygenase-2 (PDB id: 3LN1) and tumor necrosis factor-alpha (PDB id: 2AZ5). The docking methodology developed in AutoDock Vina was validated by re-docking all the co-crystallized ligands with their corresponding proteins. In addition, the docked poses of each ligand were compared with their crystal conformations, which exhibited a root-mean-square deviation (RMSD) of 2.0 A°, and validated the dependability of the docking approach used [30,32,33]. The docking results are shown in Figure 4 and Figure 5A–D and Table 2 and Table 3.

## 4. Discussion

*A. indicum* is an extensively utilized and accepted traditional medicinal plant. Early findings on the leaf extracts suggested antibacterial, hepatoprotective and larvicidal capabilities, and anti-inflammatory activity [11,37,38,39]. The study was conducted on a plant native to the area where the study took place, and which is also part of the local diet. Consequently, the present research focused on isolating and characterizing two new phytocompounds and three well-known aliphatic ester glucoside compounds that may have chemotaxonomic significance. The isolated phytoconstituents were screened for possible anti-inflammatory and analgesic therapeutic potential. Further molecular docking was used to clearly understand the analgesic and anti-inflammatory properties of *A. indicum*. Docking is an essential strategy for developing computerized medications for particular illnesses. Additionally, the online prediction tool ADME analysis was used to determine the pharmacokinetics, drug-likeness, and physicochemical properties of all isolated phytoconstituents. The structural profiling of isolated phytoconstituents from *A. indicum* methanolic extract yielded two novel phytocompounds, identifying 5′-hydroxyhexyl n-hexadecanoate as AB-01 and n-octanoyl-β-D-glucopyranosyl-(2′-1′′)-β-D-glucopyranoside as AB-05. The three previously recognized phytocompounds were also isolated as ester glucoside. Because the exact purity of the isolated compounds was not determined, it is possible that a small fraction of molecules contributed to the observed bioactivities, which might be recognized as a limitation of this study.

**Scheme 1:**
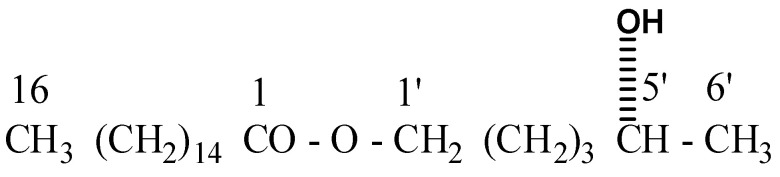
Isolated compound 5′-hydroxyhexyl n-hexadecanoate (AB 01).

**Figure 6:**
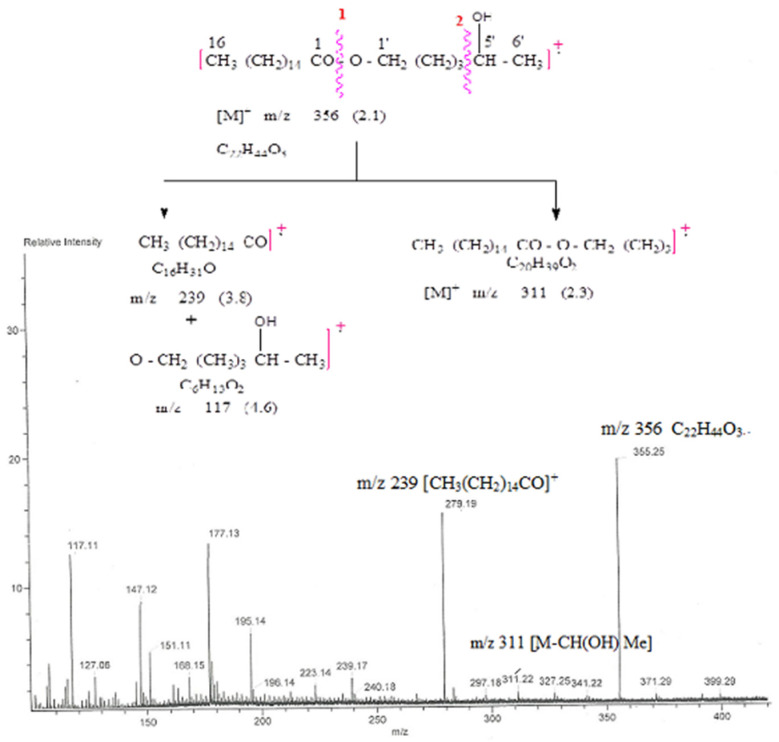
The diagnostic MS/MS fragmentation at *m/z* 792 full ESI-MS scan of the isolated compound 5′-hydroxyhexyl n-hexadecanoate.

**Compound AB-01**, Eluants from chloroform: methanol (19:1), a colorless fatty acid ester called AB-01, was produced. Its IR spectra (Figure S1) showed absorption bands for the hydroxyl group (3410 cm^−1^) and the ester function (1722 cm^−1^). The mass spectrum (Figure S2) at *m/z* 356, displayed a molecular ion peak, identical to a fatty acid ester’s chemical formula, C_22_H_44_O_3_. Hexadiol was used to esterify palmitic acid, as evidenced by the ion signal at *m/z* 239 [CH_3_(CH_2_)_14_CO]^+^ and 117 [M-239]^+^. One hydroxyl group present at C-5′ (Figure 6) was supported by the ion fragment produced at *m/z* 311 [M-CH(OH) Me]. Compound 1 ^1^H NMR spectrum (Supplementary Materials) (Figure S3) showed a one-proton broad multiplet at δ 3.64 with a half-width of 11.2 Hz attributed to the proton of β-oriented H-5′ carbinol. Two triplets of δ 3.50 (J = 7.2 Hz) and 2.56 (J = 7.5 Hz), each integrating for two protons, produced oxygenated methylene H2-1′ and methylene H2-2 next to the ester carbon, respectively. The remaining methylene protons showed up as a broad singlet at δ 1.28 (28H) and multiplets at 1δ 1.55 (2H) and 1.32 (4H). A six-proton multiplet accounted for the terminal C-16 and C-6′ methyl protons at a value of 0.88. Compound AB-01’s ^13^C NMR spectra (Figure S4) showed indications for ester carbon at δ 173.41 (C-1), carbinol carbon at δ 73.90 (C-5′), Methylene carbons at δ 64.39 (C-1′), δ 38.32 to 22.48 (all oxygenated), and δ 18.61 (C-1′) and 14.21 (both oxygenated) (C-14). These arguments led to the formulation of compound AB-01’s chemical structure as 5′-hydroxyhexyl n-hexadecanoate (Scheme 1). It was identified as a label aliphatic ester.

**Scheme 2:**
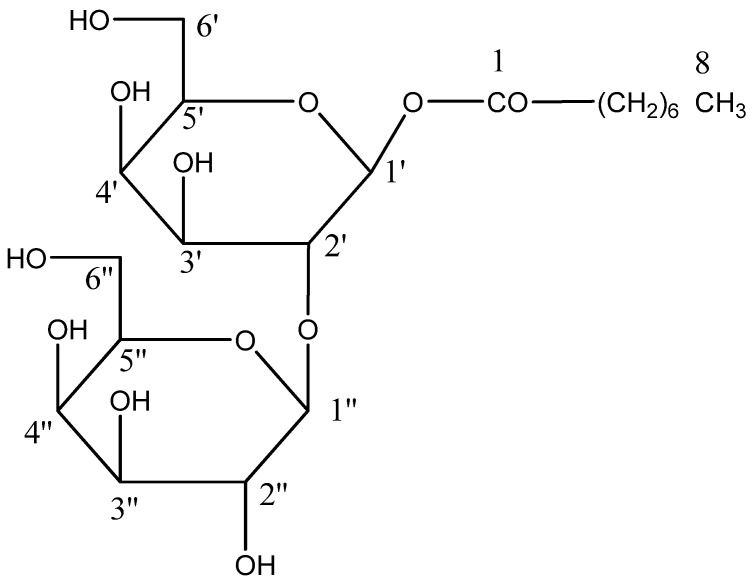
Isolated compound n-octanoyl-β-D-glucopyranosyl-2′-β-D-glucopyranoside (AB 02).

**Figure 7:**
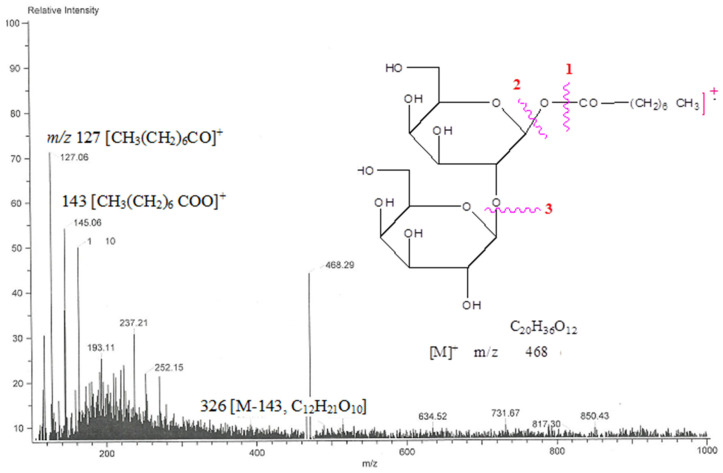
The diagnostic MS/MS fragmentation at *m/z* 792 full ESI-MS scan of the isolated compound n-octanoyl-β-D-glucopyranosyl-2′-β-D-glucopyranoside.

From chloroform: methanol (19:1) eluants, the crystalline mass of chemical AB-02 capryloyl diglucoside was synthesized. It demonstrated IR absorption bands (Figure S5) for hydroxyl groups (3450, 3389, 3265 cm^−1^), ester function (1721 cm^−1^), and aliphatic chain (779 cm^−1^) and produced a positive Fehling solution for glucoside. The molecular ion peak of chemical AB-02 (Figure S6) was identified at *m/z* 468 based on mass and ^13^C NMR spectra, which is compatible with the molecular formula of acyl diglycoside, C_20_H_36_O_12._ The ion peaks arising at *m/z* 127 [CH_3_(CH_2_)_6_CO] ^+^ and 143 [CH_3_(CH_2_)_6_ COO]^+^ indicated that aglycone capric acid was esterified with the diglycosidic glycone unit. The ion fragments generating at *m/z* 163 [C_6_H_11_O_5_]^+^ and 326 [M-143, C_12_H_21_O_10_] suggested that the aglycone unit was connected to the C12 sugar unit (Figure 7). Compound 2 ^1^H NMR spectra (Figure S7) revealed two one-proton doublets at δ 4.04 (J = 7.2 Hz) and 3.99 (J = 7.1 Hz), which were attributed to the anomeric protons 1 + -1′ and H-1′′, respectively. Between δ 3.83–3.10, the other sugar protons made an appearance. Methylene H2-2 near the ester function was found to have a two-proton triplet at around δ 2.56 (J = 7.2 Hz). The acyl unit’s methylene protons were responsible for a broad singlet at δ 1.29 and a two-proton multiplet at δ 1.39 (8H). The terminal C-8 primary methyl protons were attributed to a three-proton triplet at about δ 0.88 (J = 6.3 Hz). Compound 2’s ^13^C NMR spectra (Figure S8) showed signals for methyl carbon at 14.21δ 14.21(C-8), ester carbon at δ 172.06 (C-1), and anomeric carbons at δ 103.29 (C-1′) and 99.33 (C-1′′), as well as additional sugar carbons between δ 34.82 and 22.63. The saturated nature of the molecule was corroborated by the lack of any signal in the 1H NMR spectrum above δ 4.04 and the 13C NMR spectrum between δ 172.06 and 130.29. The second sugar unit may have been located at C-2′, as suggested by the presence of C-2′ in the deshielded area at δ 83.41 in the ^13^C NMR spectra. Capric acid D-glucose was produced via the compound AB-02’s acid hydrolysis (TLC comparable). The structure of component AB-02 was determined as n-octanoyl-β-D-glucopyranosyl-2′-β-D-glucopyranoside (Scheme 2) based on these proofs.

**Scheme 3:**
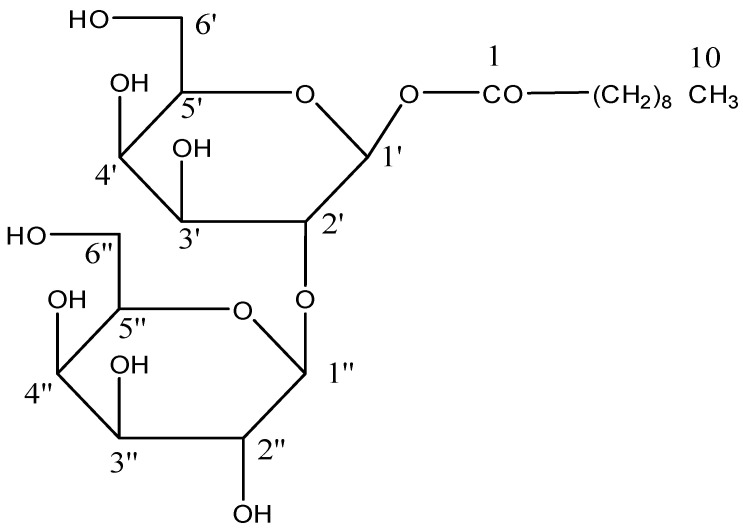
Isolated compound n-deconoyl-β-D-glucopyranoryl-(2′→1′′)-β-D-glucopyranoside (AB 03).

**Figure 8:**
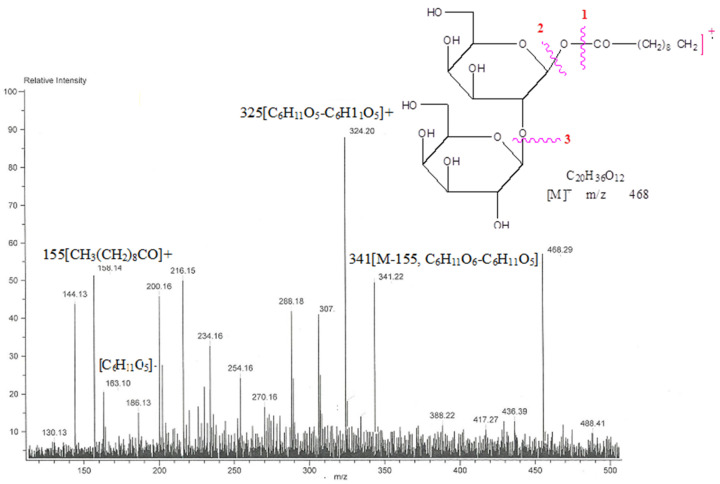
The diagnostic MS/MS fragmentation at *m/z* 792 full ESI-MS scan of the isolated compound n-deconoyl-β-D-glucopyranoryl-(2′→1′′)-β-D-glucopyranoside.

Chloroform: methanol yielded capryl diglucoside, molecule AB-03, as a colorless crystalline mass (9:1). It accepted the glycoside Fehling solution test and displayed IR absorption bands (Figure S9) for hydroxyl groups (3425, 3389, 3261 cm^−1^) and ester groups (1721 cm^−1^), as well as aliphatic chains (779 cm^−1^). The compound 3 molecular ion peak (Figure S10) was identified at *m/z* 497 [M+H]+, which, according to its mass and ^13^C NMR spectra, is an acyl glycoside with the formula C_22_H_41_O_2_. The ion peaks at *m/z* 155[CH_3_(CH_2_)_8_CO]^+^ and 341[M-155, C_6_H_11_O_6_-C_6_H_11_O_5_], 325[C_6_H_11_O_5_-C_6_H_11_O_5_]^+^ and 163 [C_6_H_11_O_5_]^-^ showed the existence of a caproyl group as an aglycone unit connected to a disaccharide chain (Figure 8). Two one-proton doublets at δ 4.06 (J = 7.3 Hz) and 4.01 (J = 7.1 Hz) in the ^1^H NMR spectra of compound 3 (Figure S11) were attributed to anomeric H-1′ and H-1′′ protons, respectively. Between δ 3.83-3.30, the other sugar protons started to show themselves. The proton of methylene H2-2, which is close to the ester group, was attributed to a two-proton doublet at around δ 2.23 (J = 7.2 Hz). A broad singlet and a two-proton multiplet of the other sugar proton were seen at δ 1.29 (12H) and 1.52, respectively. Three protons in a triplet at about δ 0.86 (J = 6.3 Hz) were determined to be primary methyl protons from C-10. Compound 3 ^13^C NMR spectra (Figure S12) showed indications for ester carbon at δ 170.16 (C-1), anomeric carbons at δ 103.27 (C-1), and 99.33 (C-1′′), respectively, methylene carbon ranges from δ 34.91 to 22.61, methyl carbon at δ 14.23 (C-8), and other sugar carbon ranges from δ 83.39 to 64.33. The saturated nature of the molecule was corroborated by the lack of any signal in the ^1^H NMR spectrum above δ 4.06 and the ^13^C NMR spectrum between δ 170.16 and 103.27. The connection of the sugar unit at C-2′ was suggested by the presence of the C-2′ signal in the deshield area at δ 83.39 in the ^13^C NMR spectrum. Compared to co-TLC, compound AB-03’s acid hydrolysis produced palmitic acid and D-glucose. On the analysis of chemical processes and spectral data, compound 3’s chemical structure was identified as n-deconoyl-β-D-glucopyranoryl-(2′→1′′)-β-D-glucopyranoside (Scheme 3).

**Figure 9:**
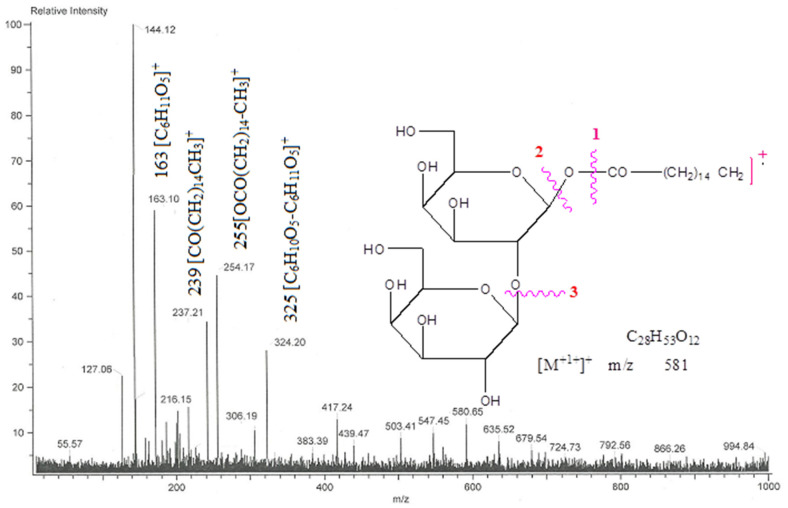
The diagnostic MS/MS fragmentation at *m/z* 792 full ESI-MS scan of the isolated compound AB-04.

**Scheme 4:**
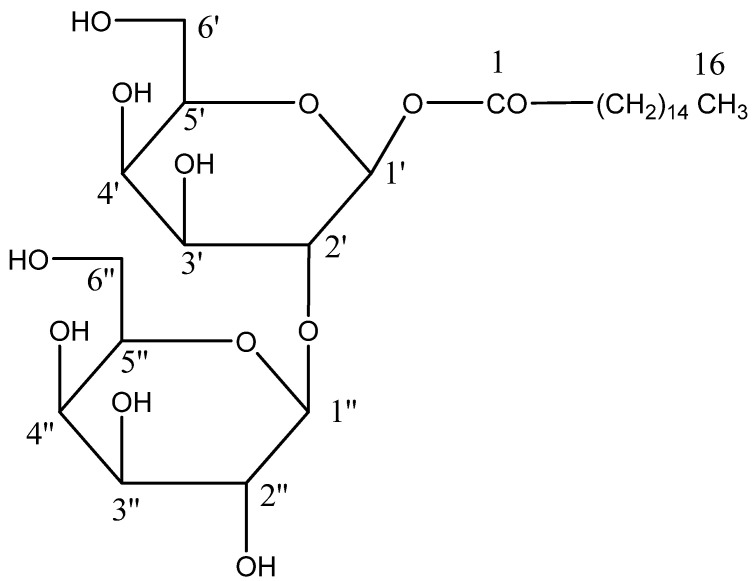
The isolated compound n-hexadecanoyl-(2′→1′′)-β-D-glucopyranosyl-β-D-glucopyranoside (AB-04).

Chloroform and methanol (9:1) eluants were used to separate compound AB-04, palmityl diglucoside, a colorless crystalline mass. After carrying out a Fehling solution test, glycosides were revealed to be present, as well as IR absorption bands (Figure S13) for hydroxyl (3448, 3356, 3251 cm^−1^), ester (1725 cm^−1^), and aliphatic chain (779 cm^−1^). According to its molecular formula C28H53O12, compound AB-04’s molecular ion peak was discovered at *m/z* 581[M+H], based on mass (Figure S14) and ^13^C NMR spectra. The ion peaks appearing at *m/z* 239 [CO(CH_2_)_14_CH_3_]^+^, 255[OCO(CH_2_)1_4_-CH_3_]^+^, 325 [C_6_H_10_O_5_-C_6_H_11_O_5_]^+^ and 163 [C_6_H_11_O_5_]+ showed that the diglycoside unit had a palmityl chain connected to it (Figure 9). Two one-proton doublets at δ 4.04 (J = 7.2 Hz) and 4.01 (J = 7.0 Hz) in compound AB 04 ^1^H NMR spectra (Figure S15) were attributed to anomeric H-1′ and H-1′′ protons, respectively, with an additional sugar proton from δ 3.96 to 3.30, and a two-proton triplet at δ 2.31 (J = 7.2 Hz) attributable to the methylene H_2_-2 proton next to the ester group. Additional methylene protons, including a two-proton multiplet at δ 1.62, a singlet at δ 1.29 (24H), and three proton triplets at δ 0.88 (J = 6.2 Hz), were responsible for the C-16 main methyl protons. Signals for ester carbons at 171.52 (C-1), anomeric carbons at 103.27 (C-1) and 93.32 (C-1), other sugar carbons within δ 83.39 to 62.90, methylene carbons from δ 33.88 to δ 22.55, and methyl carbon at δ 14.61 were seen in compound AB 04 ^13^C NMR spectra (C-16). The attachment of the second sugar unit at C-2′ was suggested by the existence of the C-2′ carbon signal in the deshielded area at δ 83.39. No signal was seen after δ 4.04 in the ^1^H NMR spectrum or from δ 171.52 to 103.27 in the ^13^C NMR spectrum (Figure S16), indicating that the molecule was already saturated. Compound AB-04 acid hydrolysis generated palmitic acid and glucose, which were equivalent when analyzed using thin-layer chromatography (TLC). Based on this evidence, the chemical structure of compound 4 was determined as n-hexadecanoyl-(2′→1′′)-β-D-glucopyranosyl-β-D-glucopyranoside (Scheme 4).

**Figure 10:**
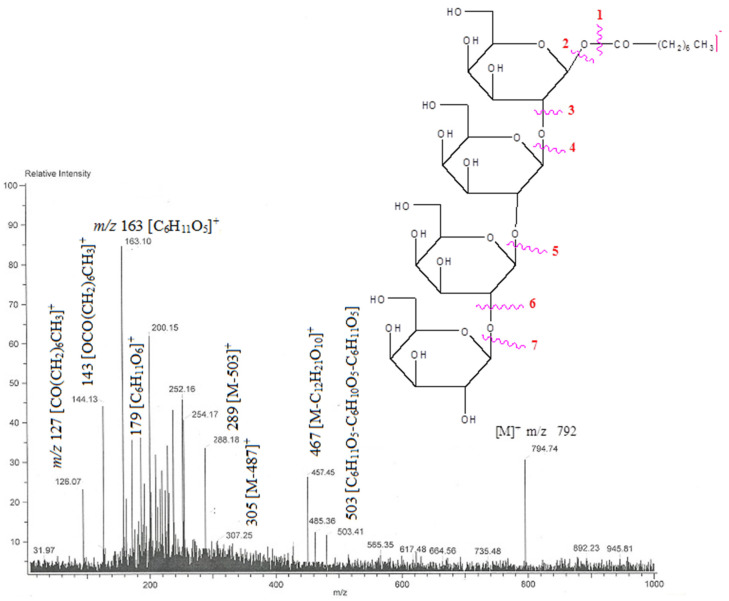
The diagnostic MS/MS fragmentation at *m/z* 792 full ESI-MS scan of the isolated compound AB-05.

**Scheme 5:**
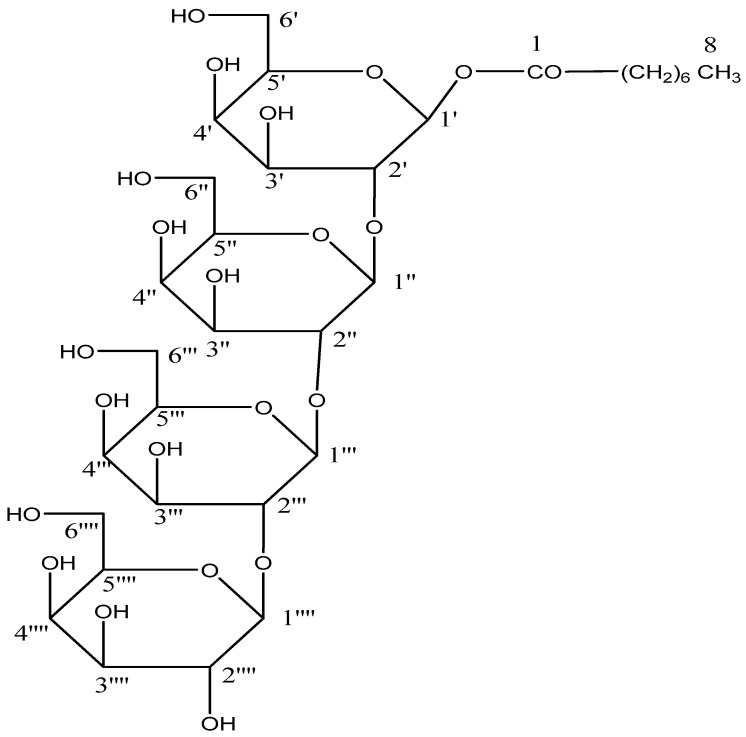
Isolated compound (n-octanoyl-β-D-glucopyranosyl-(2′-1′′)-β-D-glucopyranosyl-(2′′-1′′′)-β-D-glucopyranosyl- (2′′′-1′′′′)- β-D-glucopyranoside) (AB-05).

The AB-05 compound capryloyl tetra glucoside was a light brown crystal mass produced from an eluant of chloroform-methanol (9:1). It showed positive glycoside tests. The hydroxyl groups (3425, 3397, 3265 cm^−1^), IR absorption bands (Figure S17), as well as ester groups (1723 cm^−1^), and aliphatic chains were visible (779 cm^−1^). Based on the mass (Figure S18) and ^13^C NMR spectra, it was determined that the peak of the molecular ion for compound AB-05 was located at 792 [M]+. This result was in accordance with the chemical formula of the acyl tetraglycoside, which is C_32_H5_6_O_2_. It was determined that the capryl group was connected to a tetra glycoside unit based on the ion peaks that occurred at *m/z* 127 [CO(CH_2_)_6_CH_3_]^+^ and 143 [OCO(CH_2_)_6_CH_3_]^+^. The *m/z* 163-generated ion fragments 467 [M-C_12_H_21_O_10_]+, 305 [M-487]+, 289 [M-503]+, 487 [C6H_11_O_5_-C_6_H_10_O_5_-C_6_H_11_O_5_]+ 503 [C_6_H_11_O_5_-C_6_H_10_O_5_-C_6_H_11_O_5_], 179 [C_6_H_11_O_6_]+, [C_6_H_11_O_5_]^+^. The binding of a tetraglycosidic unit to a capryloyl group was facilitated by 629 [M-163]+ (Figure 10). The ^1^H NMR spectra of compound AB 05 (Figure S19) exhibited four one-proton doublets at δ 4.06 (J = 7.1 Hz), 4.04 (J = 7.2 Hz), 4.02 (J = 7.0 Hz), and 4.00 (J = 7.3 Hz). These doublets were assigned to the anomeric H-1′, H-1′′, H-1′′′ and H-1′′′′ protons, respectively. Other sugar protons ranged in frequency from δ 3.96 to 3.29, methylene protons ranged in frequency from δ 2.51 to 1.25, and a triplet of three protons at δ 0.86 (J = 6.3 Hz) was attributed to C-8 primary methyl protons. Compound AB 05 13C NMR spectra (Figure S20) showed indications for ester carbon at δ 171.13 (C-1), anomeric carbon at δ 105.89 (C-1′), 103.20 (C-1′), 99.33 (C-1′′′), and 98.16 (C-1′′′′), among other locations, δ 84.24 to 62.62 for additional sugar protons, methylene carbons at δ 33.13, 29.86, and 22.84, and methyl carbon at δ 14.27 (C-8). The deshielded portion of the 13C NMR spectrum’s presence of C-2′ (at δ 84.24) and C-2′′, C-2′′′ (at δ 83.18) implied (2→1) connections of the sugar units. The ^13^C NMR spectrum showed no signal between δ 171.13 and 105.89 and no signal beyond δ 4.06 in the ^1^H NMR spectrum, indicating that the molecule was saturated. Based on these evidences, compound AB-05’s structure was clarified as n-octanoyl-β-D-glucopyranosyl-(2′-1′′)-β-D-glucopyranosyl-(2′′-1′′′)-β-D-glucopyranosyl- (2′′′-1′′′′)- β-D-glucopyranoside (Scheme 5). This is an entirely new capryloyl tetra-glucoside. All the compounds AB 01-AB 05, IR, Mass ^1^H-NMR, and ^13^C NMR spec-trum are provided in the supplementary material file.

Figure 1 displays the anti-inflammatory effects of isolated phytoconstituents AB-01 to AB-05 at 5 and 10 mg/kg and Indomethacin (10 mg/kg) on carrageenan-induced rat paw. When carrageenan-stimulated rats were given Indomethacin in combination with AB-01, AB-02, AB-03, AB-04, and AB-05 at a dosage of 10 mg/kg, there was a statistically significant reduction (*p* < 0.05) in the volume of their paws. Compared to a higher dose of Indomethacin (10 mg/kg), the effects of AB-01 and AB-05 at 10 mg/kg were shown to be more effective in lowering the volume of the swollen paw. The aggravation is linked to the pathophysiology of different diseases, such as joint inflammation, cancerous growths, gout, and vascular diseases. In traditional clinical frameworks, various medicinal plants treat pain and inflammation signs. We found that at 5.0 and 10.0 mg/kg dosages, the AB-01, AB-02, AB-03, AB-04, and AB-05 extracted from *A. indicum* extract had a calming and analgesic effect, respectively. Carrageenan acted as a phlogistic factor, increasing the synthesis of prostaglandins and bradykinins at certain periods [40].

All the drugs that were examined demonstrated a reduction in paw edema volume from 1 h to 5 h, and their anti-inflammatory effect was continued beyond 3 h. This analysis unequivocally showed that all examined medications may have had an effect that cooperated with the prostaglandins surge. Curiously, the effects of AB-05 on carrageenan-induced paw edema were consistent throughout the 1 h and 5 h time points. Inflammatory mediators such as prostaglandins and platelet-activating factors (PAF) were triggered by carrageenan, leading to inflammation. Histamine, 5-hydroxytryptamine, and kinin all arrived during the first stage (0–1 h), whereas the second stage (3–5 h) was linked to the production of prostaglandin and bradykinin. Even though the 10 mg/kg dose had a greater calming effect than the 0.05 mg/kg dose, this finding was directly connected to the effects shown in carrageenan-induced paw edema, where both dosages of isolated compounds markedly changed the inflammatory response triggered by carrageenan [41]. The abdominal constrictions seen following the acetic acid treatment were related to the prostaglandin-induced sensitization of nociceptive receptors in assessing analgesic potential. Therefore, potential consequences of the concentrates may exert their pain-relieving effects by impeding prostaglandins synthesis.

In the ADMET studies, QPlogHERG values for compounds AB 01 and AB 04 were less than the specified value, indicating they possess some cardiotoxic potential. Predictions of the Qikprop (version 5.1, Schrödinger, LLC, New York, NY, 2020) tool suggested that the non-active transport of these compounds across the gut-blood barrier was poor except for compound AB 01, with complete oral absorption. This was also the only compound capable of crossing the blood-brain barrier, as suggested by its QPlogBB and QPlogMDCK values. All compounds had some degree of skin permeability. As per Lipinski’s rule of five, compound AB 01 exhibited only one violation; compounds AB 02 and AB 03 showed two violations, while compounds AB 04 and AB 05 displayed three out of five (Table 1). Hence, all data obtained from the predictions of the Qikprop tool gave an idea that compound AB 01 might serve the purpose of a potentially druggable compound. The isolated compounds AB-02, AB-03, AB-04, and AB-05 were docked with several receptors linked to inflammatory pathways. These receptors included cyclooxygenase-2 and tumor necrosis factor-alpha. Comparing the docked poses to the crystal conformation of each ligand revealed a root-mean-square deviation (RMSD) of 2.0 A°, further establishing the reliability of the selected docking method.

## 5. Conclusions

The phytochemical components of *A. indicum*, collected from the banks of the Koshi River in India, were depicted in this study. As a result, this study can significantly advance our understanding of the phytoconstituents in this herb and pave the way for the creation of novel medications with enormous promise for use in traditional Indian medicine. The current investigation suggests that AB-01, AB-03, and AB-05 show considerable analgesic and anti-inflammatory effects at 10 mg/kg. The main trigger for the release of platelet initiating factors (PAF), other inflammatory mediators and prostaglandins was carrageenan-induced edema. In conclusion, 5′-hydroxyhexyl n-hexadecanoate as AB-01 and n-octanoyl-β-D-glucopyranosyl-(2′-1′′)- β-D-glucopyranoside as AB-05 were isolated from *A. indicum* extract. These compounds showed anti-inflammatory and pain-relieving properties and may help to treat systemic inflammation caused by endotoxin. Since the precise purity of the extracted compounds has not yet been established, a potential contribution of minor compounds to the reported bioactivities cannot be completely ruled out. To the best of our knowledge, this work is the first to examine the presence of capryloyl tetra-glucoside and aliphatic ester in a methanolic extract of *A. indicum*. The study confirms that these strong isolated chemicals can be a useful diagnostic tool for the genus *A. indicum.* These results are thought to be crucial in nurturing the powerful compounds AB-01 and AB-05 for treating disorders caused by inflammation.

## Figures and Tables

**Table 1:** Data obtained from ADMET prediction.

Compound	AB 01	AB 02	AB 03	AB 04	AB 05
Mol. Wt.	356.591	468.497	496.551	580.712	792.781
Rot bond	18	18	20	26	30
H-bond donor	1	7	7	7	13
H-bond acceptor	3.7	19	19	19	36
Dipole	1.712	4.111	1.514	3.253	3.36
QPlogPo/w	5.922	−2.055	−1.487	0.334	−6.255
QPlogHERG	−5.85	−4.589	−4.972	−5.887	−4.01
QPPCaco (nm/s)	1193.933	25.694	26.629	25.667	5.006
QPlogBB	−1.723	−3.359	−3.617	−4.479	−4.523
QPPMDCK (nm/s)	599.178	9.453	9.825	9.442	1.272
QPlogKp	−1.599	−5.655	−5.697	−4.852	−7.971
Percent Human Oral Absorption	100	15.69	19.382	17.071	0
Rule of Five	1	2	2	3	3

**Table 2:** Estimated potential energy values for the isolated molecules after energy minimization.

Compound	Initial Potential Energy (kcal/mol)	Potential Energy (kcal/mol)	Van der Waals Energy (kcal/mol)	Electrostatic Energy (kcal/mol)
Molecule 1	−10.1874	−22.8631	−9.73178	−19.5173
Molecule 2	67.315	6.36013	−11.7357	−37.0988
Molecule 3	67.3404	5.36027	−13.6227	−36.3396
Molecule 4	68.7227	4.01368	−16.9201	−35.0438
Molecule 5	169.388	55.9117	−20.5852	−57.9615

**Table 3:** Intramolecular interactions among the extracted molecules and the active site residues of TNF-alpha.

Compound	TNF_alpha Interacting Residues	Binding Energy (kcal/mol)
Molecule 1	Pi-Sigma: TYR59. Alkyl: LEU57. Pi-Alkyl: TYR59, TYR119, TYR151.	−4.0
Molecule 2	Conventional Hydrogen Bond: HIS15, TYR59, TYR151. Carbon Hydrogen Bond: HIS15. P-i-Donor Hydrogen Bond: HIS15. Pi-Sigma: TYR119. Pi-Alkyl: TYR119.	−5.3
Molecule 3	Conventional Hydrogen Bond: HIS15, TYR59, TYR151. Pi-Donor Hydrogen Bond: HIS15. Alkyl: LEU57.	−5.1
Molecule 4	Conventional Hydrogen Bond: TYR119, GLY121, SER95. Pi-Sigma: TYR119. Pi-Alkyl: TYR59, TYR119, TYR151.	−4.7
Molecule 5	Conventional Hydrogen Bond: HIS15, TYR59, GLN149, TYR151. Carbon Hydrogen Bond: HIS15. Pi-Alkyl: TYR119, TYR151.	−5.9
307	Pi-Donor: TYR151. Pi-Pi Stacked: TYR59. Pi-Pi T-Shaped: TYR119, TYR151. Alkyl: LEU57, ILE155. Pi-Alkyl: TYR59.	−6.4

## Data Availability

The data presented in this study are available on request from the corresponding authors.

## Supplementary Materials

The following supporting information can be downloaded at https://www.mdpi.com/article/10.3390/plants11192583/s1. Figure S1 IR Spectrum of AB-01; Figure S2. Mass spectrum of 5’-Hydroxyl hexyl palmitate (AB-01); Figure S3. ^1^H NMR spectrum of 5’-Hydroxyl hexyl palmitate (AB-01); Figure S4. ^13^C NMR spectrum of 5’-Hydroxyl hexyl palmitate (AB-01); Figure S5. IR Spectrum of AB-02; Figure S6. Mass spectrum of capryloyl diglucoside (AB-02); Figure S7. ^1^H NMR spectrum of capryloyl diglucoside (AB-02); Figure S8. ^13^C NMR spectrum of capryloyl diglucoside (AB-02); Figure S9. IR Spectrum of AB-03; Figure S10. Mass spectrum of capryl diglucoside (AB-03); Figure S11. ^1^H NMR spectrum of capryl diglucoside (AB-03); Figure S12. ^13^C NMR spectrum of capryl diglucoside (AB-03); Figure S13. IR Spectrum of-04; Figure S14. Mass spectrum of palmityl diglucoside (AB-04); Figure S15. ^1^H NMR spectrum of palmityl diglucoside (AB-04); Figure S16. ^13^C NMR spectrum of palmityl diglucoside (AB-04); Figure S17. IR Spectrum of AB-05; Figure S18. Mass spectrum of capryloyl tetraglucoside (AB-05); Figure S19. ^1^H NMR spectrum of capryloyl tetraglucoside (AB-05); Figure S20. ^13^C NMR spectrum of capryloyl tetraglucoside (AB-05).
