# Supplementary material for: The Structural, Biological, and In-Silico Profiling of Novel Capryloyl Tetra-Glucoside and Aliphatic Ester Constituents from the Abutilon indicum Offers New Perspectives on the Treatment of Pain and Inflammation
Source: Plants (Basel). 2022 Sep 30;11(19):2583. doi: 10.3390/plants11192583 (PMC9570646; doi:10.3390/plants11192583)
Supplement: Supplementary file 1 [file plants-11-02583-s001.zip › plants-1856022-supplementary.pdf]

Supplementary Materials: The following supporting information can be downloaded at: www.mdpi.com/xxx/s1.

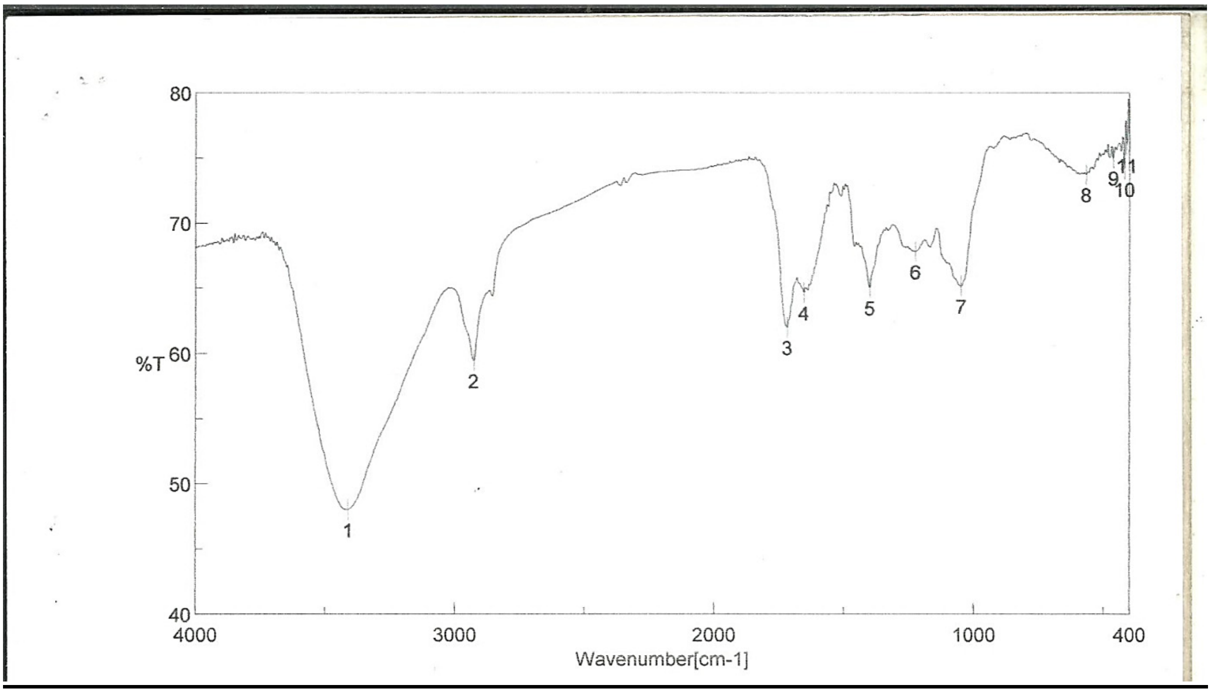

Figure S1. IR Spectrum of AB-01

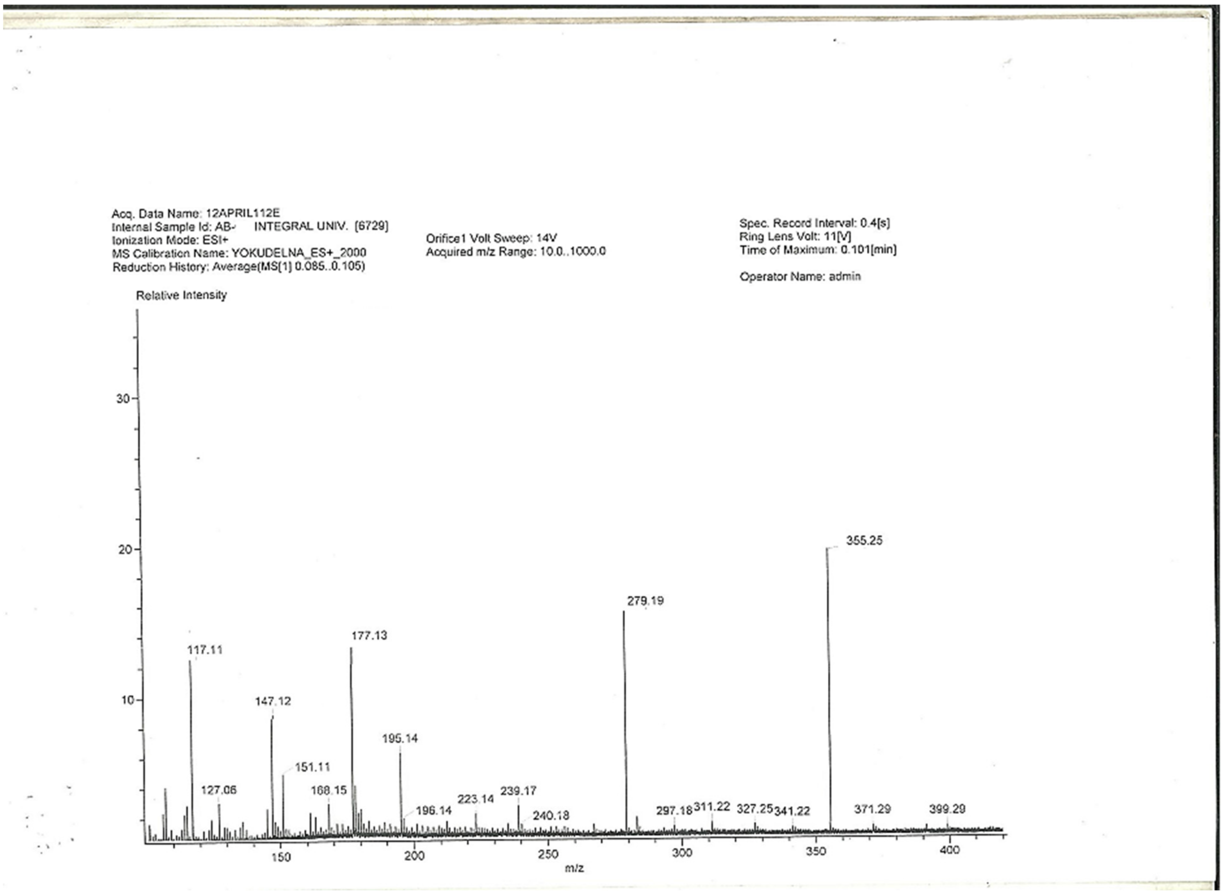

Figure S2. Mass spectrum of 5’-Hydroxyl hexyl palmitate (AB-01)

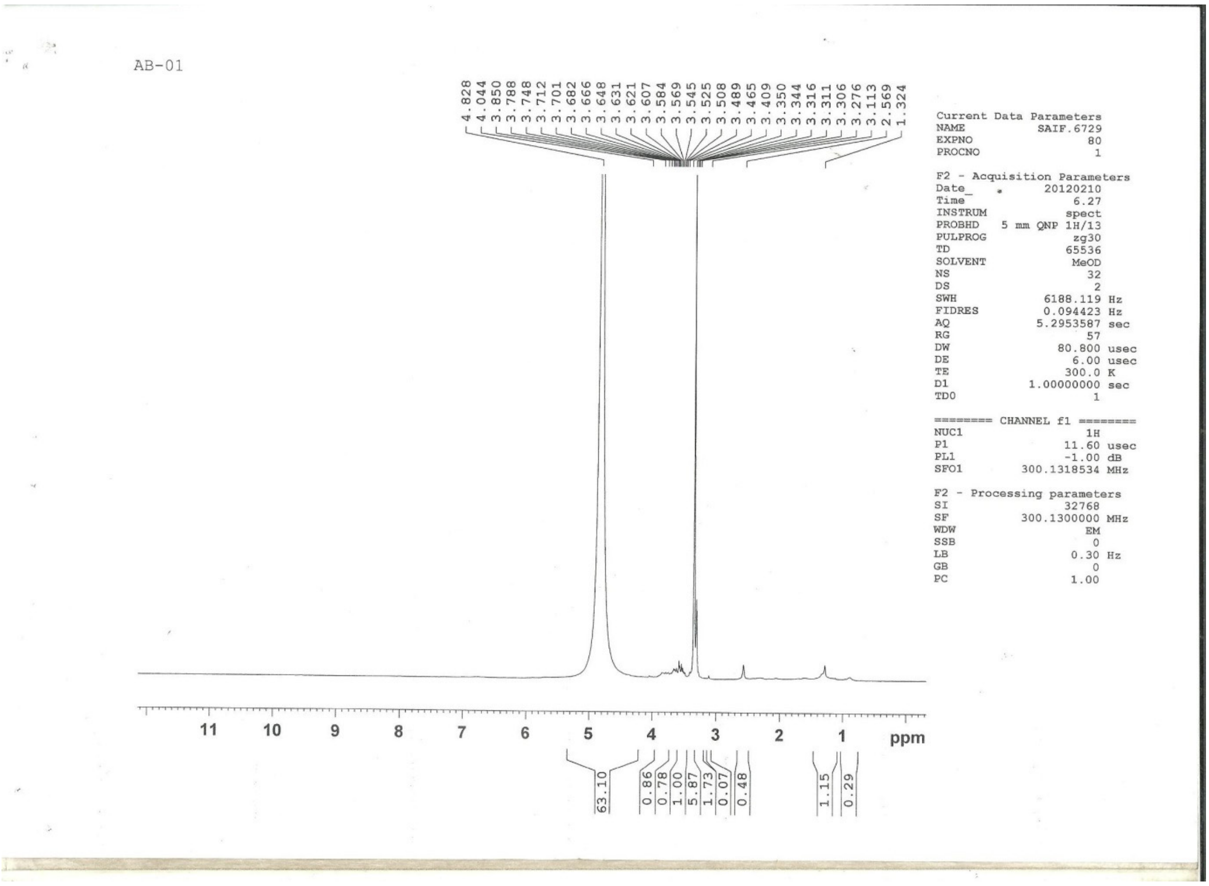

Figure S3. ^{1}H NMR spectrum of 5’-Hydroxyl hexyl palmitate (AB-01)

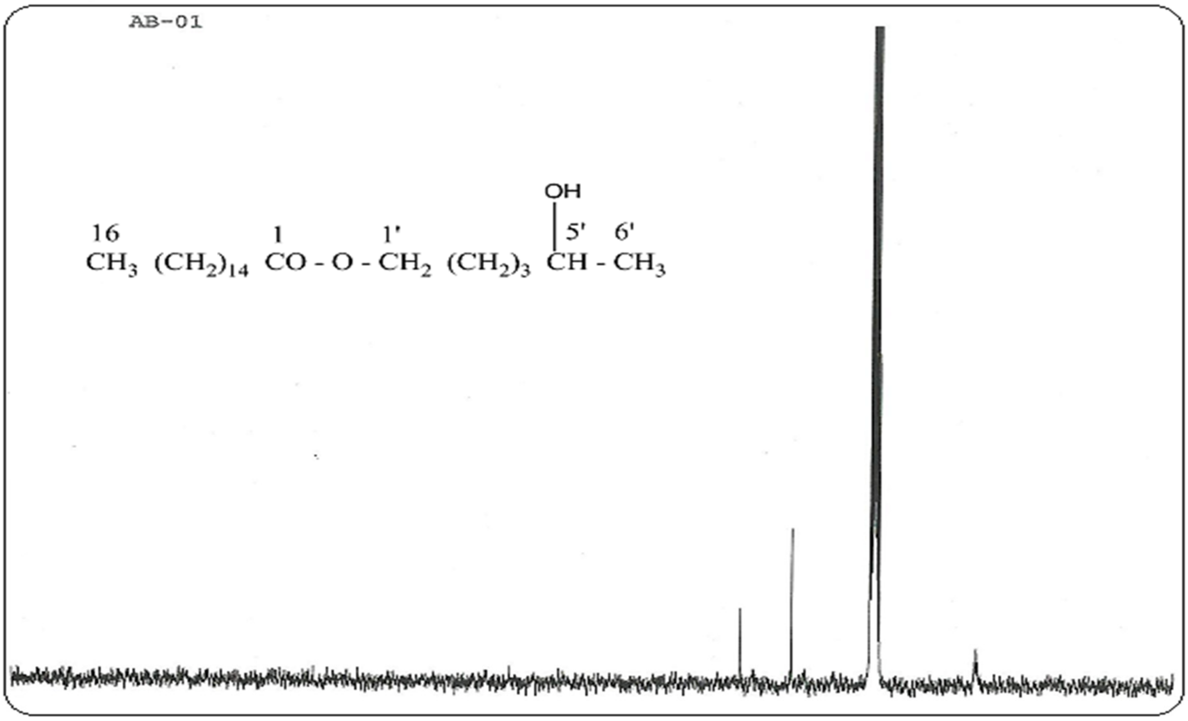

Figure S4. ^{13}C NMR spectrum of 5’-Hydroxyl hexyl palmitate (AB-01)

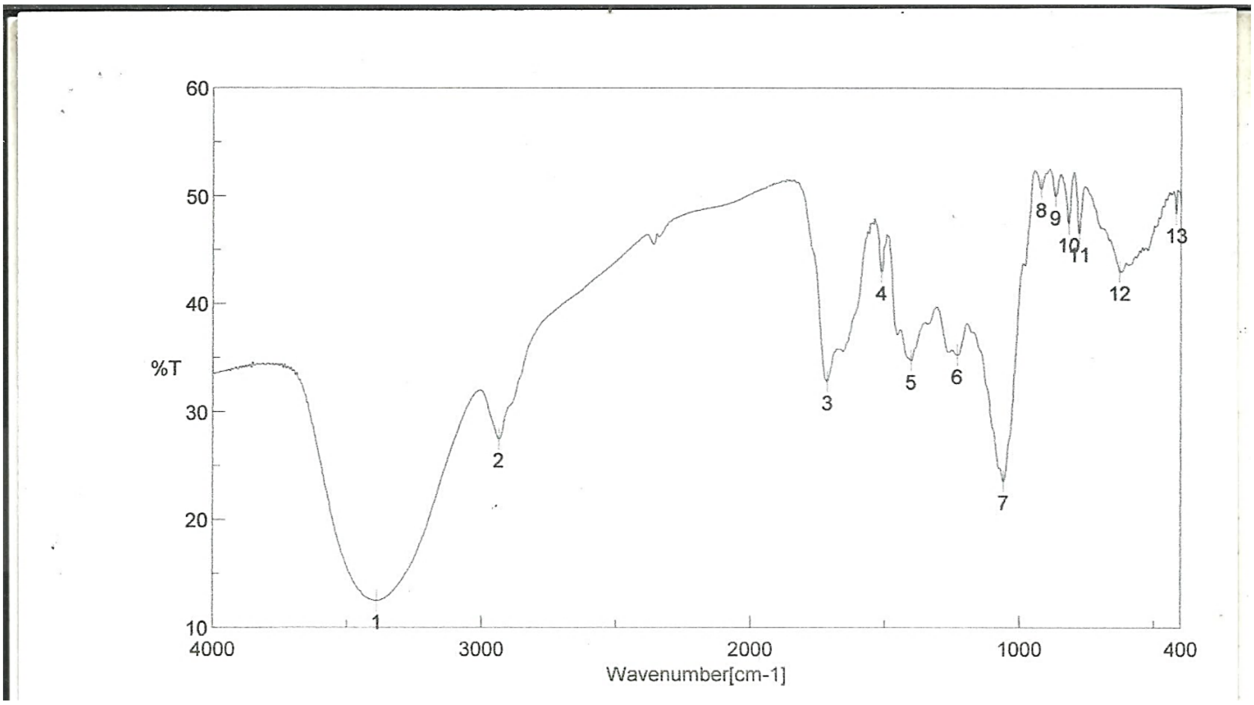

Figure S5. IR Spectrum of AB-02

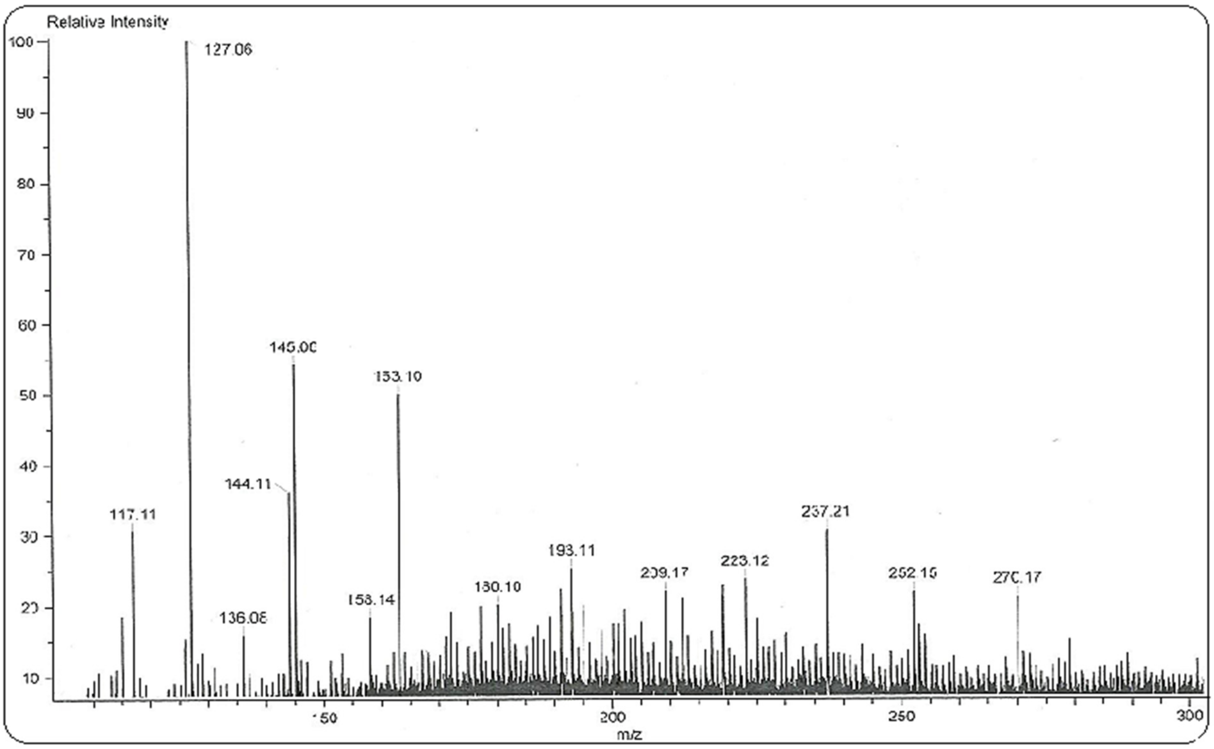

Figure S6. Mass spectrum of capryloyl diglucoside (AB-02)

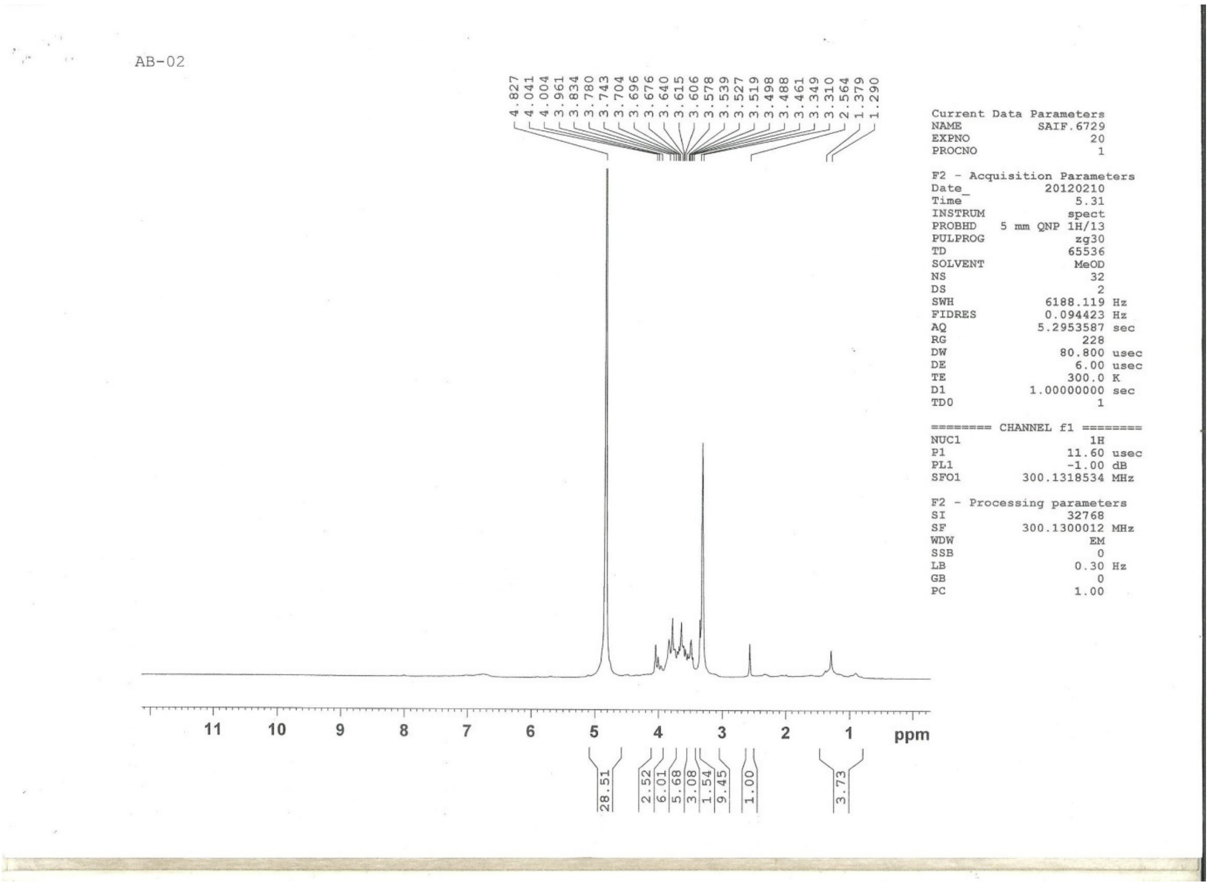

Figure S7. ^{1}H NMR spectrum of capryloyl diglucoside (AB-02)

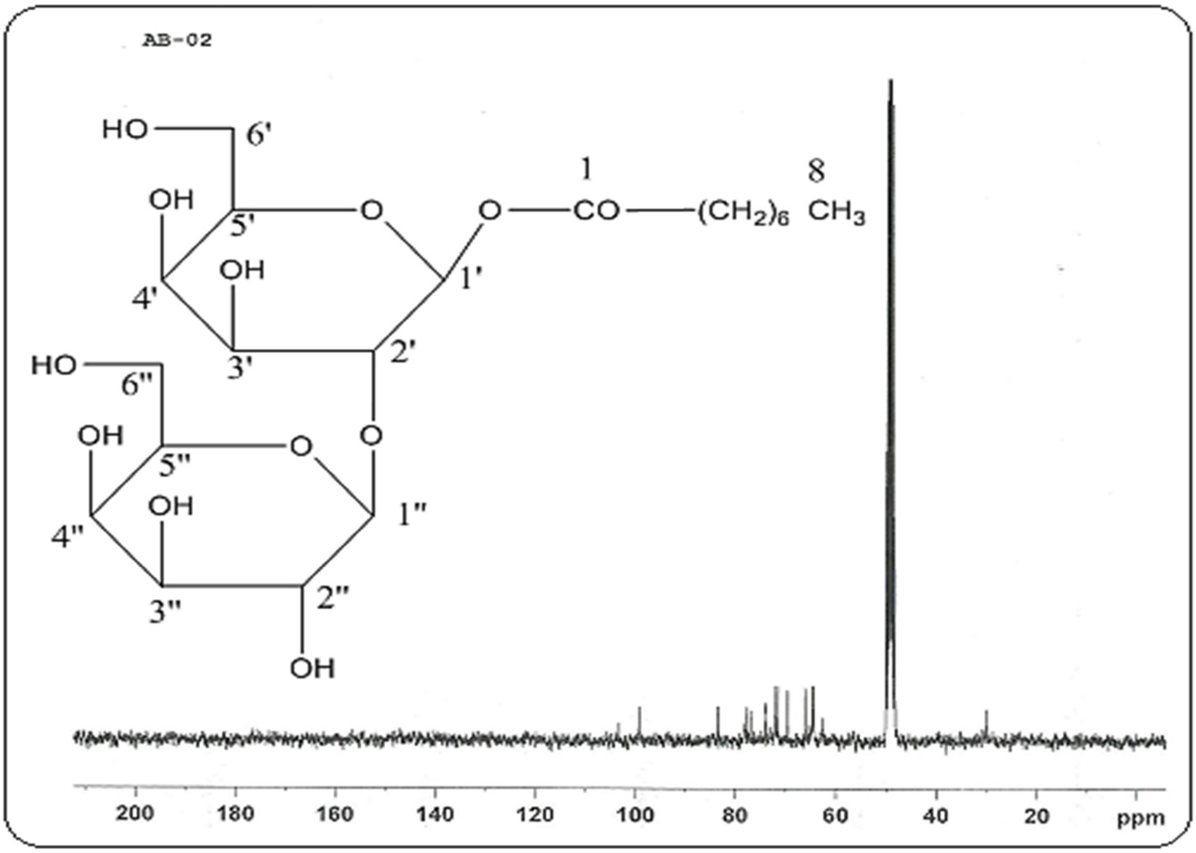

Figure S8. ^{13}C NMR spectrum of capryloyl diglucoside (AB-02)

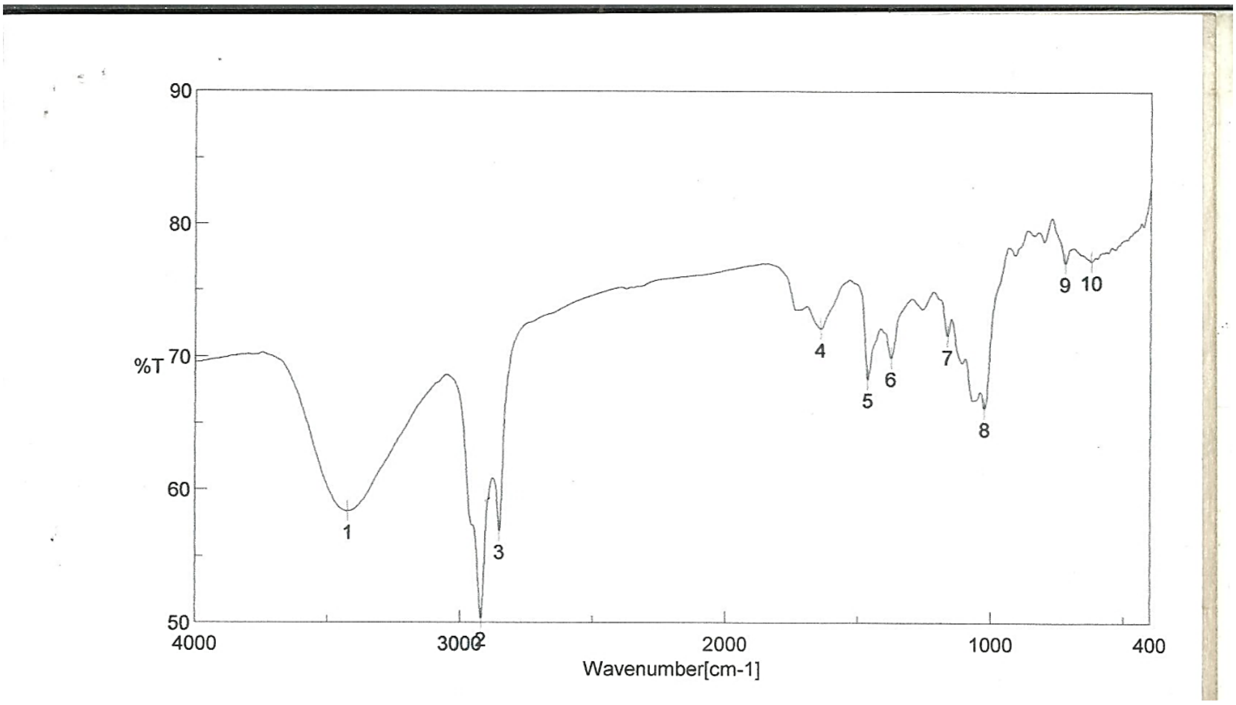

Figure S9. IR Spectrum of AB-03

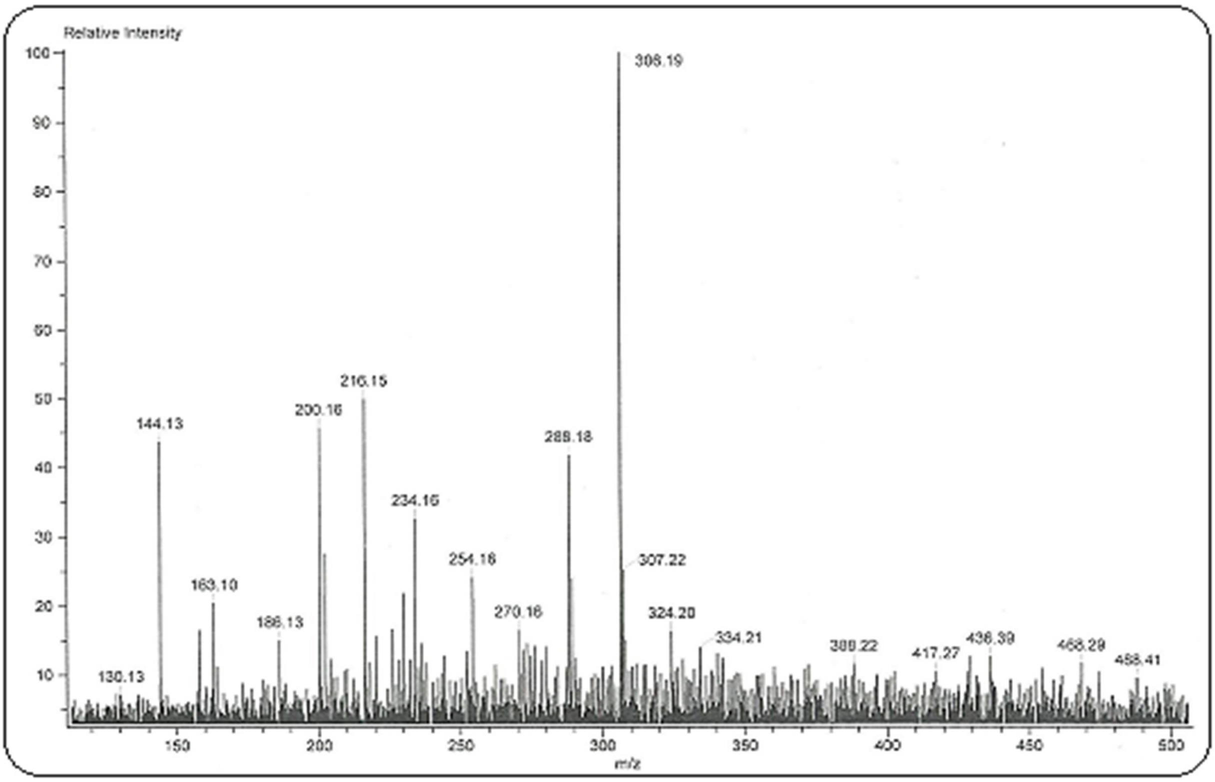

Figure S10. Mass spectrum of capryl diglucoside (AB-03)

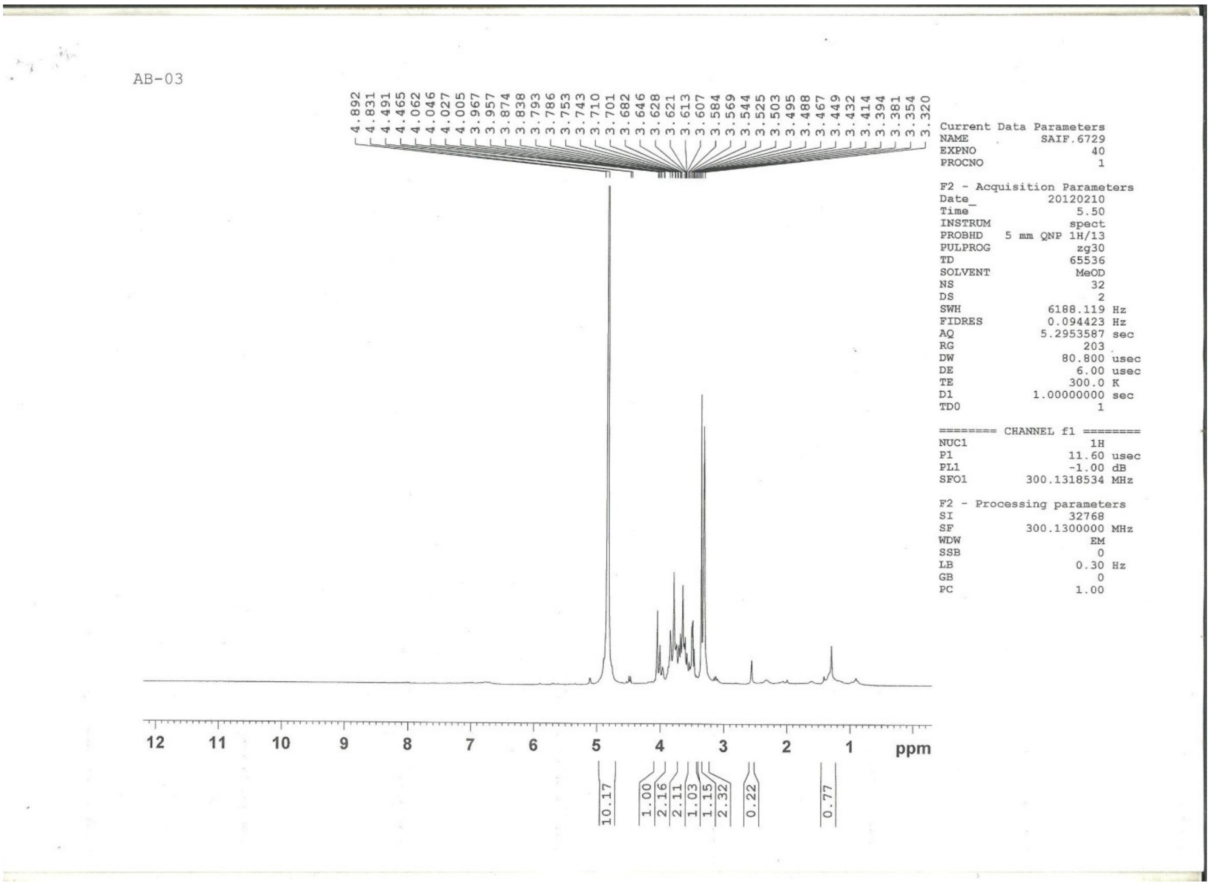

Figure S11. ^{1}H NMR spectrum of capryl diglucoside (AB-03)

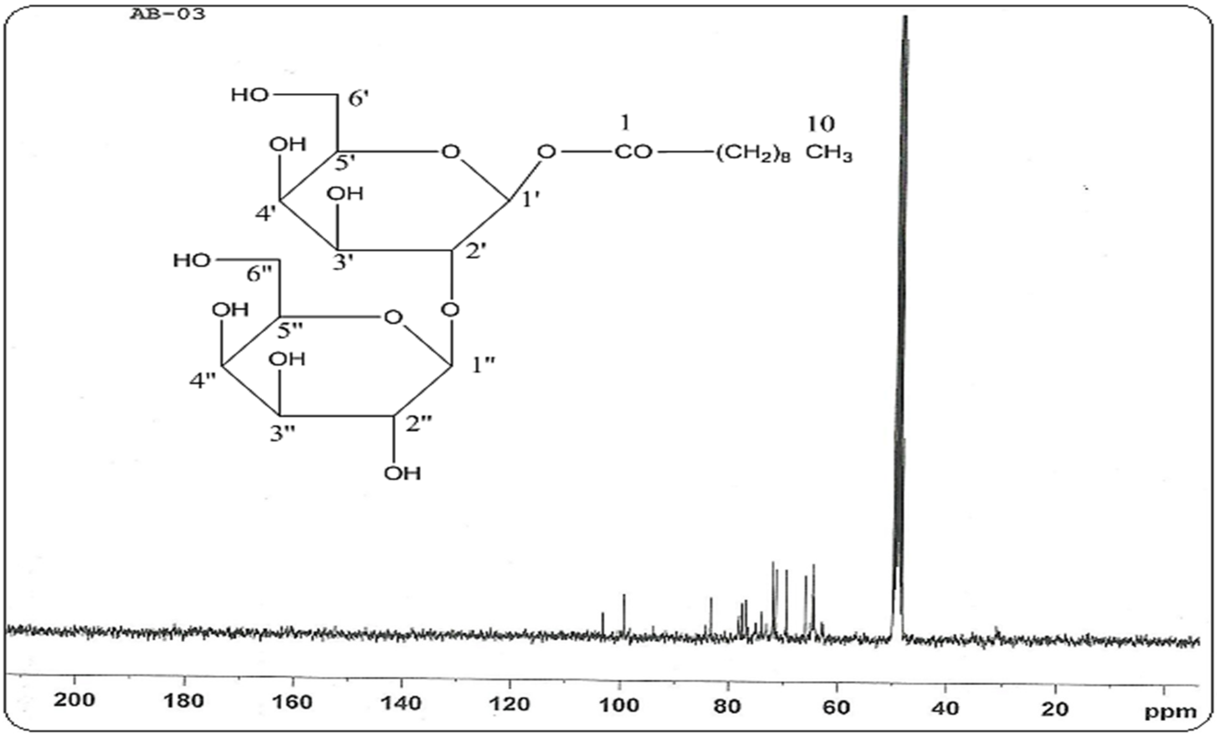

Figure S12. ^{13}C NMR spectrum of capryl diglucoside (AB-03)

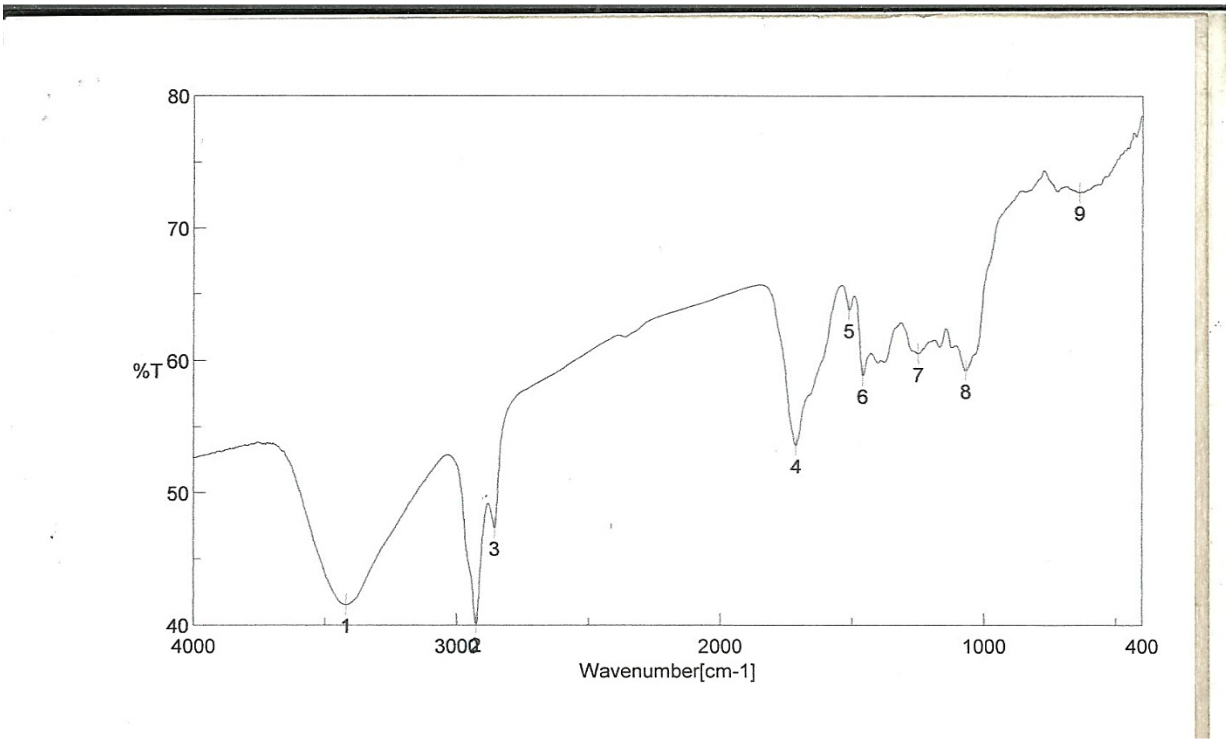

Figure S13. IR Spectrum of-04

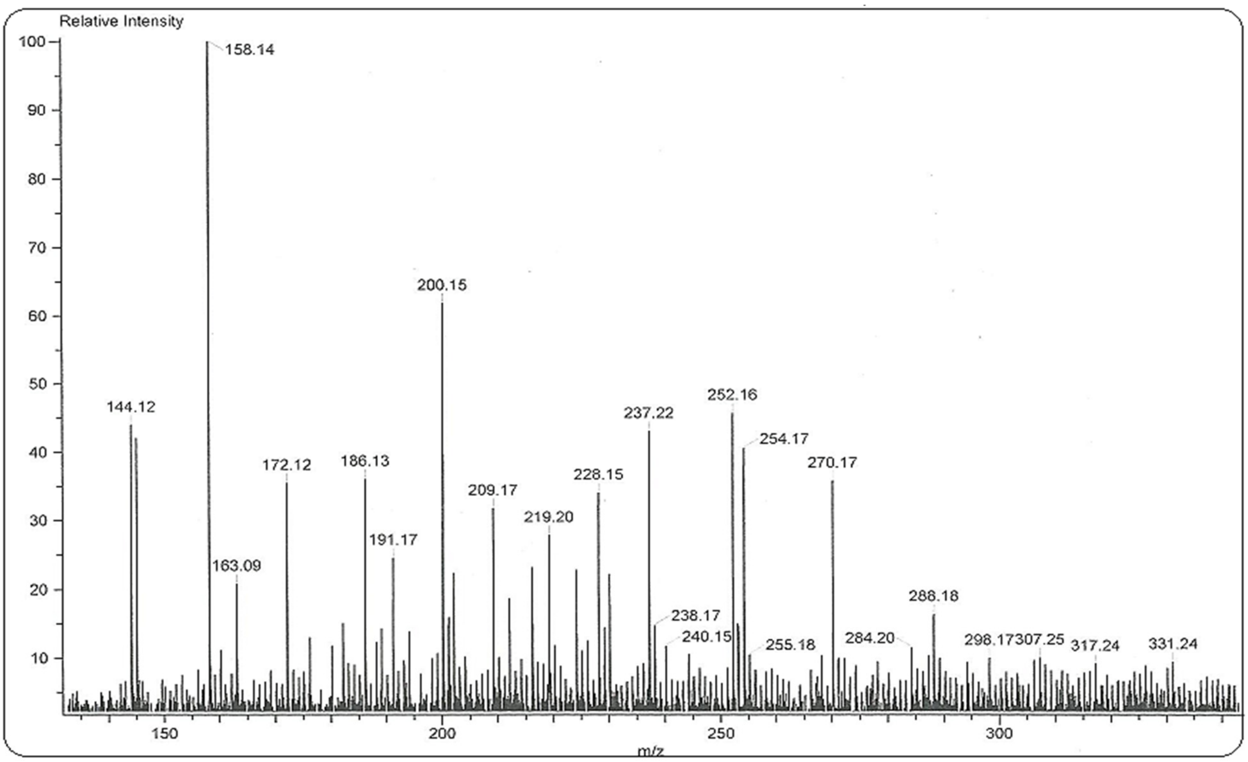

Figure S14. Mass spectrum of palmityl diglucoside (AB-04)

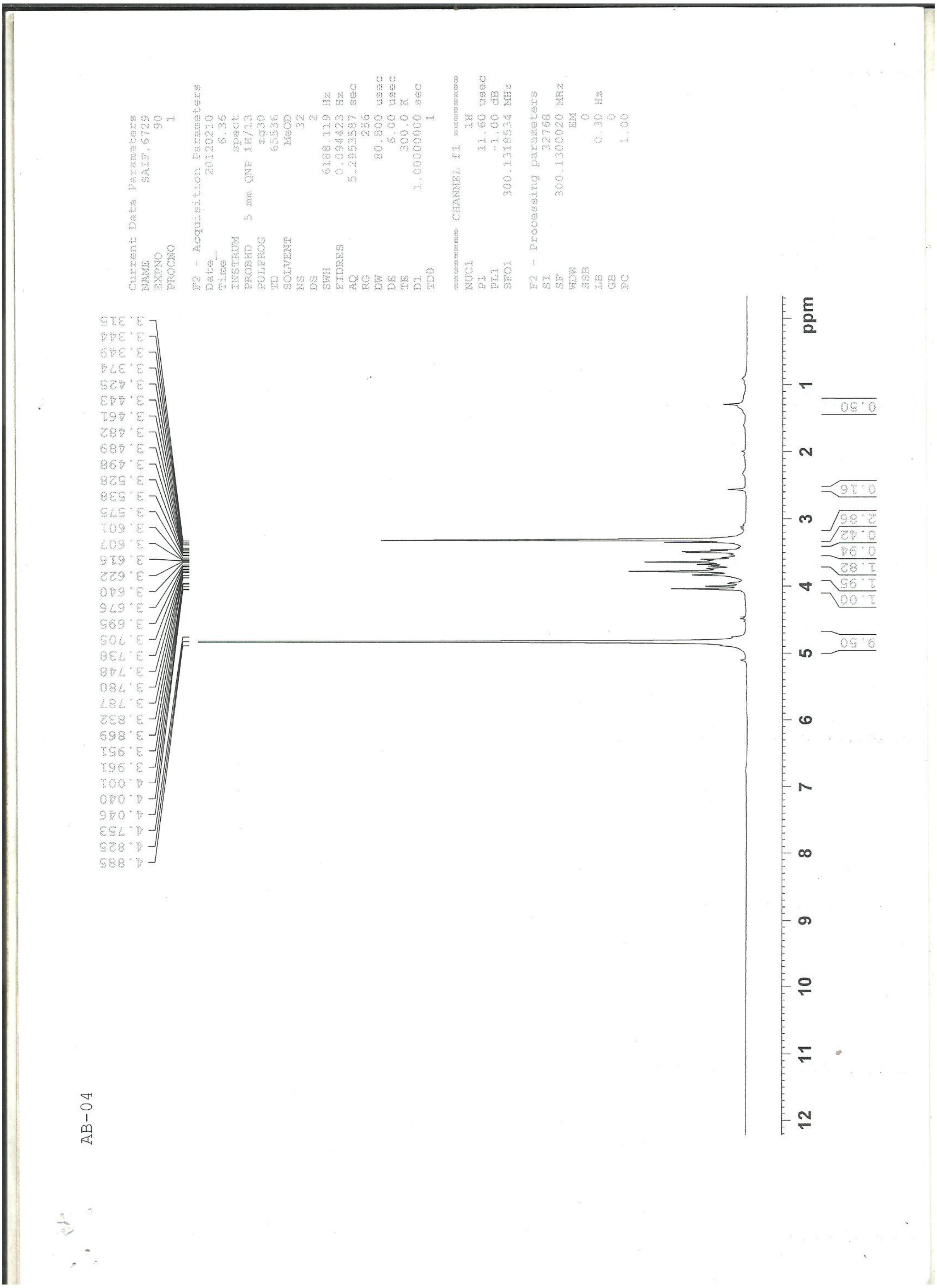

Figure S15. ^{1}H NMR spectrum of palmityl diglucoside (AB-04)

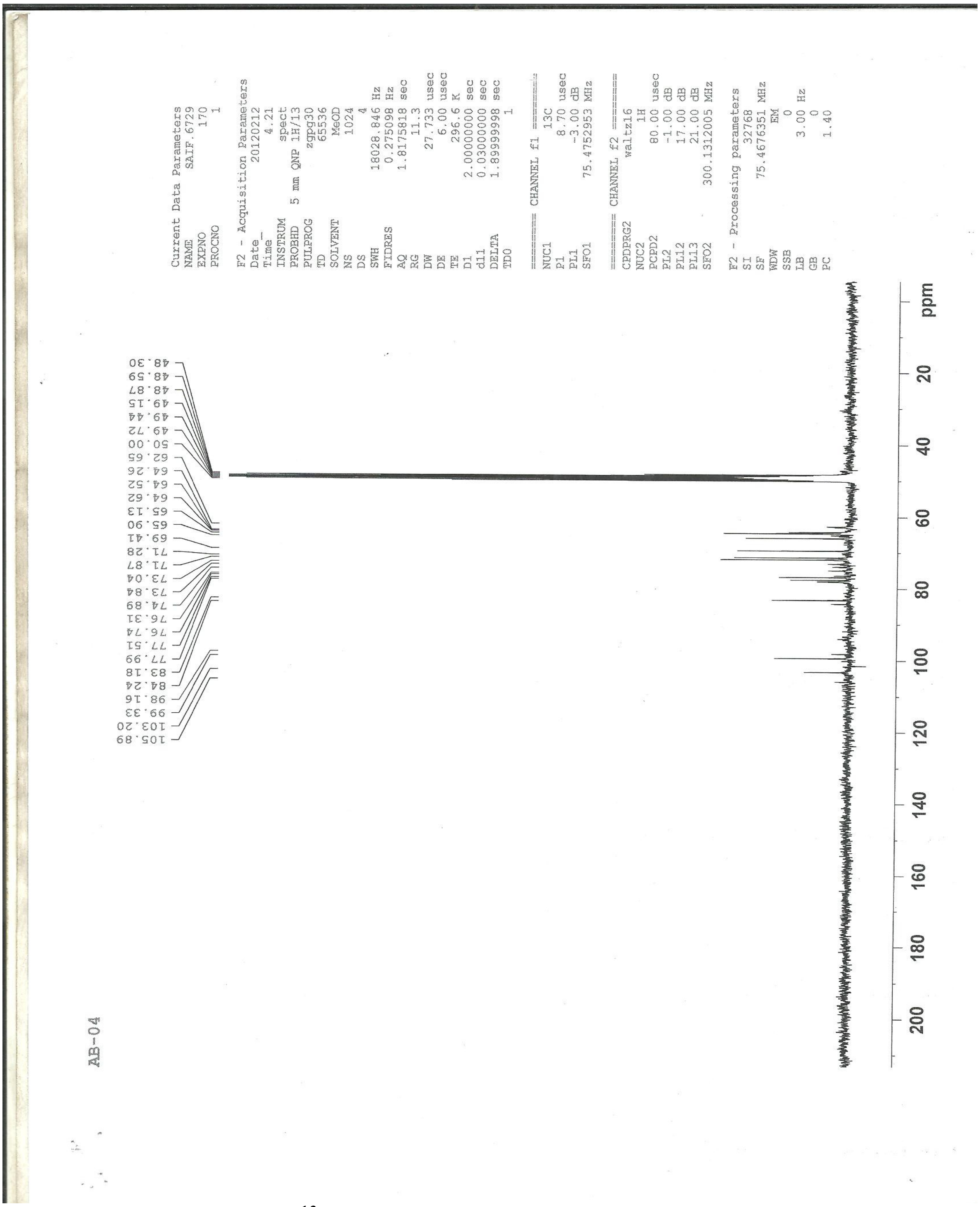

Figure S16. ^{13}C NMR spectrum of palmityl diglucoside (AB-04)

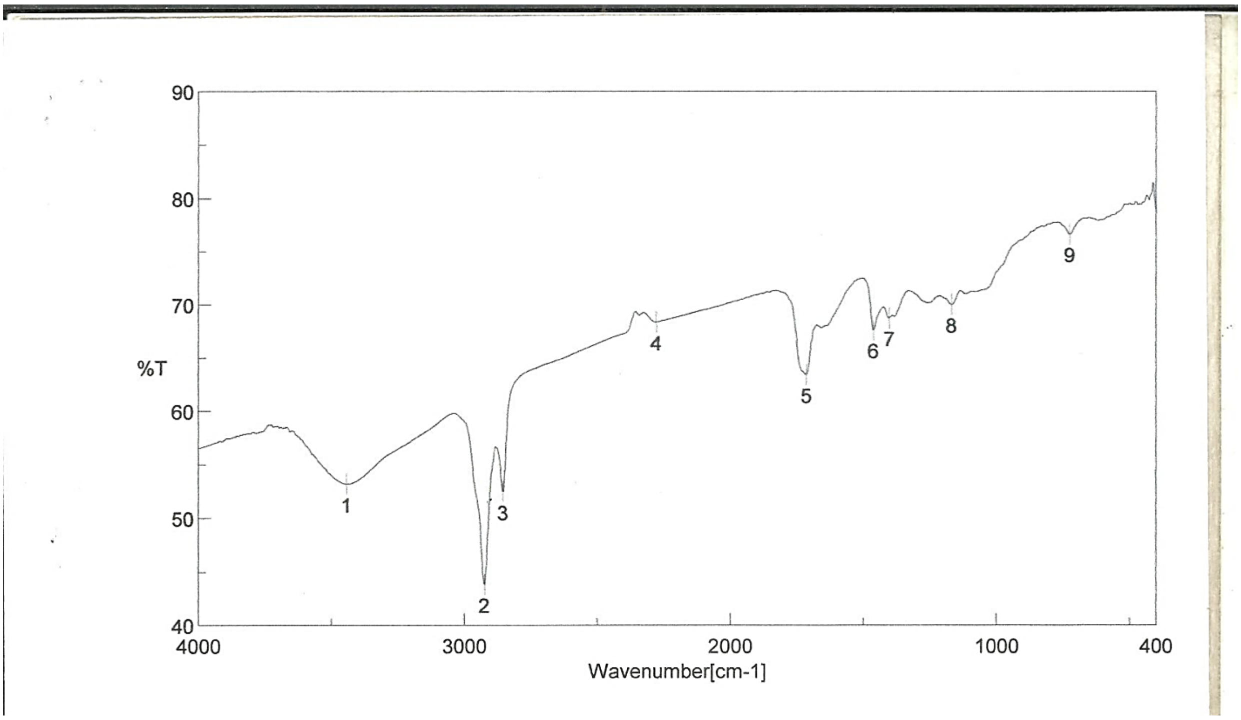

Figure S17. IR Spectrum of AB-05

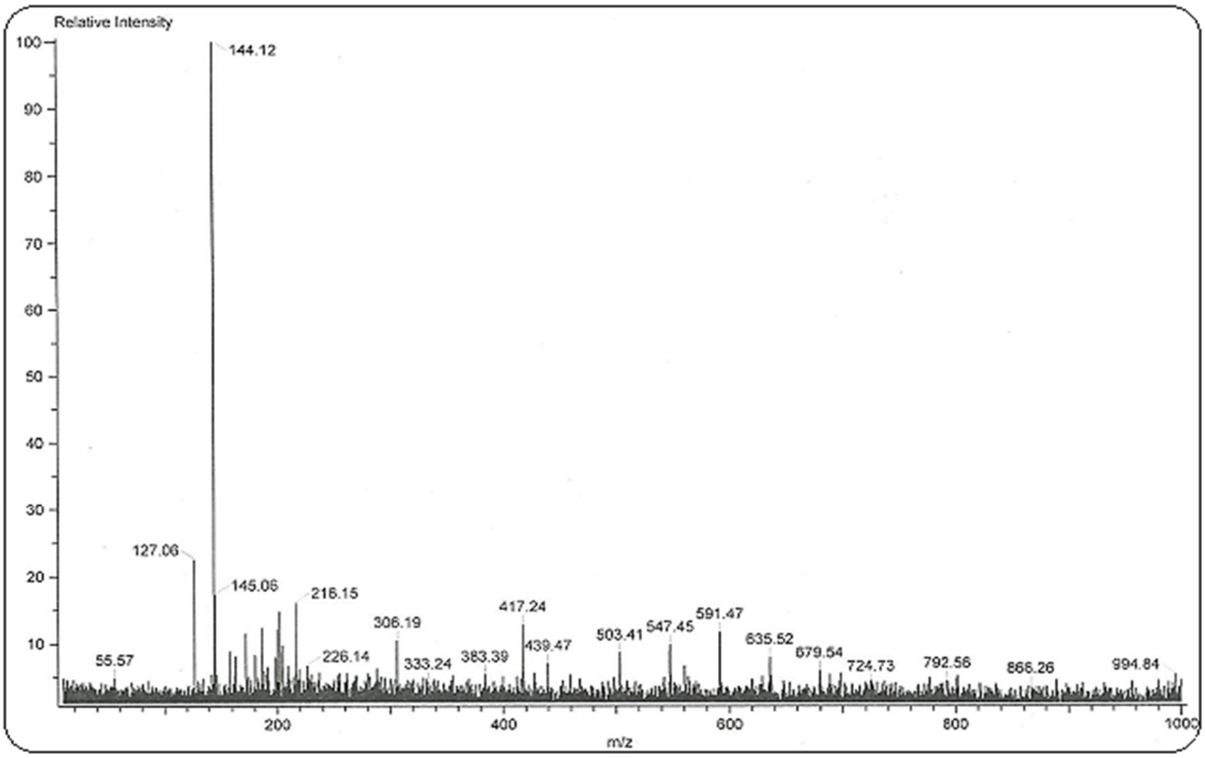

Figure S18. Mass spectrum of capryloyl tetraglucoside (AB-05)

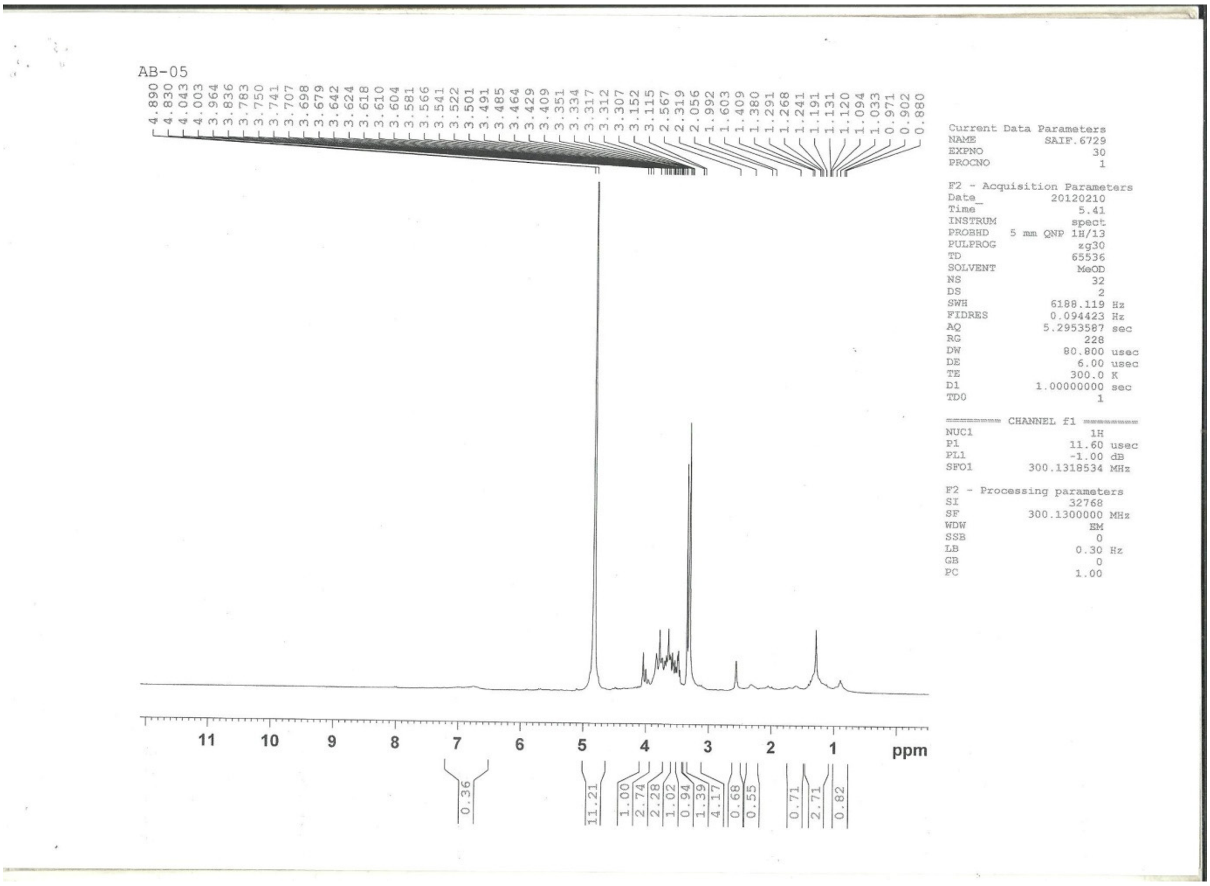

Figure S19. ^{1}H NMR spectrum of capryloyl tetraglucoside (AB-05)

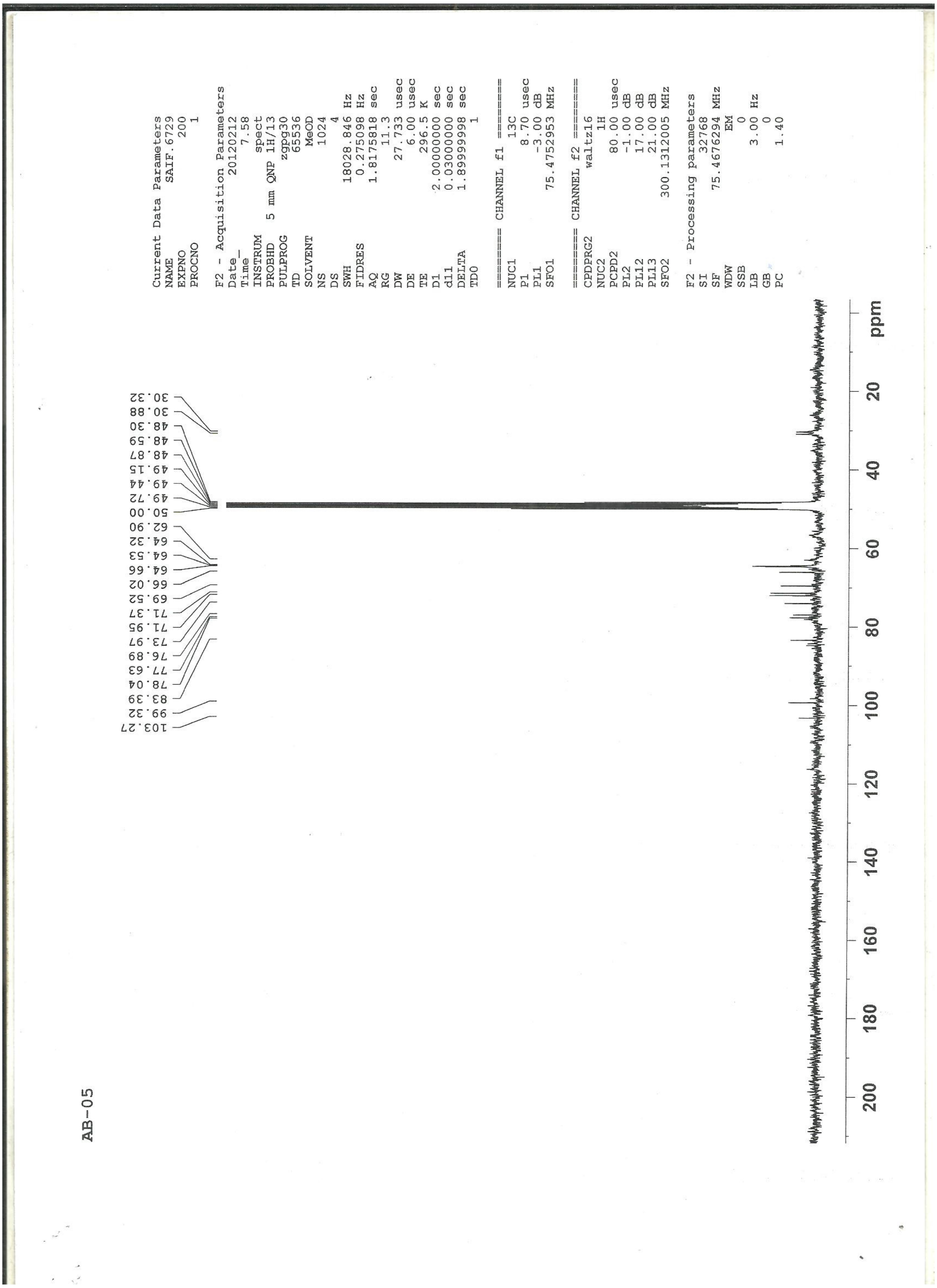

Figure S20. ^{13}C NMR spectrum of capryloyl tetraglucoside (AB-05)
